# Exploring the potential mechanism of Kaixinsan powder for the same pathogenesis of PTSD and anxiety based on network pharmacology and molecular docking: A review

**DOI:** 10.1097/MD.0000000000035869

**Published:** 2023-11-17

**Authors:** Wen-Wei Li, Jia Wang, Han-Biao Wu, Zhi-Kun Qiu

**Affiliations:** a Key Specialty of Clinical Pharmacy, The First Affiliated Hospital of Guangdong Pharmaceutical University, Guangzhou, China.

**Keywords:** anxiety, Kaixinsan powder, molecular docking, network pharmacology, PTSD, TCM

## Abstract

**Background:**

Post-traumatic stress disorder (PTSD) and anxiety are common mental illnesses and there are many similar pathogenesis and clinical manifestations between PTSD and anxiety. Kaixinsan powder (KXS), a commonly used prescription in traditional Chinese medicine, has been widely used to treat PTSD and anxiety. This study aims to explore the potential mechanisms of KXS for the same pathogenesis of PTSD and anxiety using a network pharmacology approach.

**Methods:**

The bioactive components and relevant target genes of KXS were obtained from the database about Traditional Chinese Medicine. The key genes of PTSD and anxiety were derived from disease databases. Subsequently, the network of protein–protein interaction and a network of “drug-components-disease-targets” was constructed. In order to treat PTSD and anxiety, gene ontology enrichment and signaling pathway enrichment were analyzed by using R language and components-core targets associated were validated by molecular docking.

**Results:**

One hundred three targets of KXS in treating PTSD and anxiety were identified. The results of protein–protein interaction analysis and molecular docking indicated that AKT1 and IL-6 were crucial targets. Moreover, KEGG analysis has shown that neuroactive ligand-receptor interaction, calcium signaling pathway, and cAMP signaling pathway may play crucial roles in treating PTSD and anxiety. Ten biological process, 10 molecular function, and 10 cellular component were revealed via gene ontology analysis.

**Conclusions:**

The network pharmacology study and molecular docking indicated that KXS treated anxiety and PTSD by multiple components, targets, and signaling pathways. These results provide an important reference for subsequent basic research on PTSD and anxiety.

## 1. Introduction

According to epidemiological studies in several countries, most adults will experience a traumatic event at some time in their lives, although there is variation in the prevalence of specific types of traumatic events among different countries. The clear link between post-traumatic stress disorder (PTSD) and traumatic events has been confirmed by epidemiological research.^[1,2]^ In most cases, patients with PTSD frequently occur with anxiety, mood, or substance-use disorders. It relates to serious premature death, disability, and medical illness.^[3]^ According to the Diagnostic and Statistical Manual of Mental Disorders 5, the symptoms of PTSD are classified into several clusters: intrusion/reeexperiencing, avoidance, negative cognition and mood, and hyper-arousal.^[4]^ Unfortunately, the available medication options for PTSD are limited. Only paroxetine and sertraline had been approved for treating PTSD, but the efficacy of paroxetine and sertraline was limited.^[5]^

In a recent study, anxiety disorders are estimated to range from 3.8% to 25% across countries, and estimated prevalence rates are near 70% in patients with chronic health conditions. The daily function, well-being, and quality of life of patients were impacted by anxiety seriously.^[6]^ Typical symptoms of patients with anxiety include numbness, persistent and excessive worry, recurrent panic attacks, and dizziness, etc.^[7]^ The primary medication which has been approved to treat anxiety is selective serotonin reuptake inhibitors (SSRIs), but its side effect of increasing the incidence of suicidality is unbearable.^[8]^ Due to the severe side effects and limited efficacy of medication for treating PTSD and anxiety, novel treatments and drug searching is necessary.

According to traditional Chinese medicine records, the main functions of KXS are nourishing the heart, replenishing qi and calming the mind. It is crucial for treating PTSD and anxiety and modern pharmacology has been proven that several effects are presented in KXS, including improving the ability of learning and memory, anti-anxiety, anti-fatigue, and tranquilizing the mind by multiple components acting on multiple targets. KXS has treated a variety of mental disorders, but the mechanisms of KXS are still unclear.^[9–11]^ Network pharmacology is a common method that collects bioactive components of drugs and related pathways or proteins of diseases through various databases. And then exploring the mechanisms of drugs by constructing and analyzing a “drugs-targets-diseases” network. It can not only improve the success rate of clinical trials of new drugs, but also save on drug research and development costs. This article uses network pharmacology to explore the mechanism of action of drugs, providing a theoretical basis for subsequent related research.^[12,13]^

**Figure 1.**
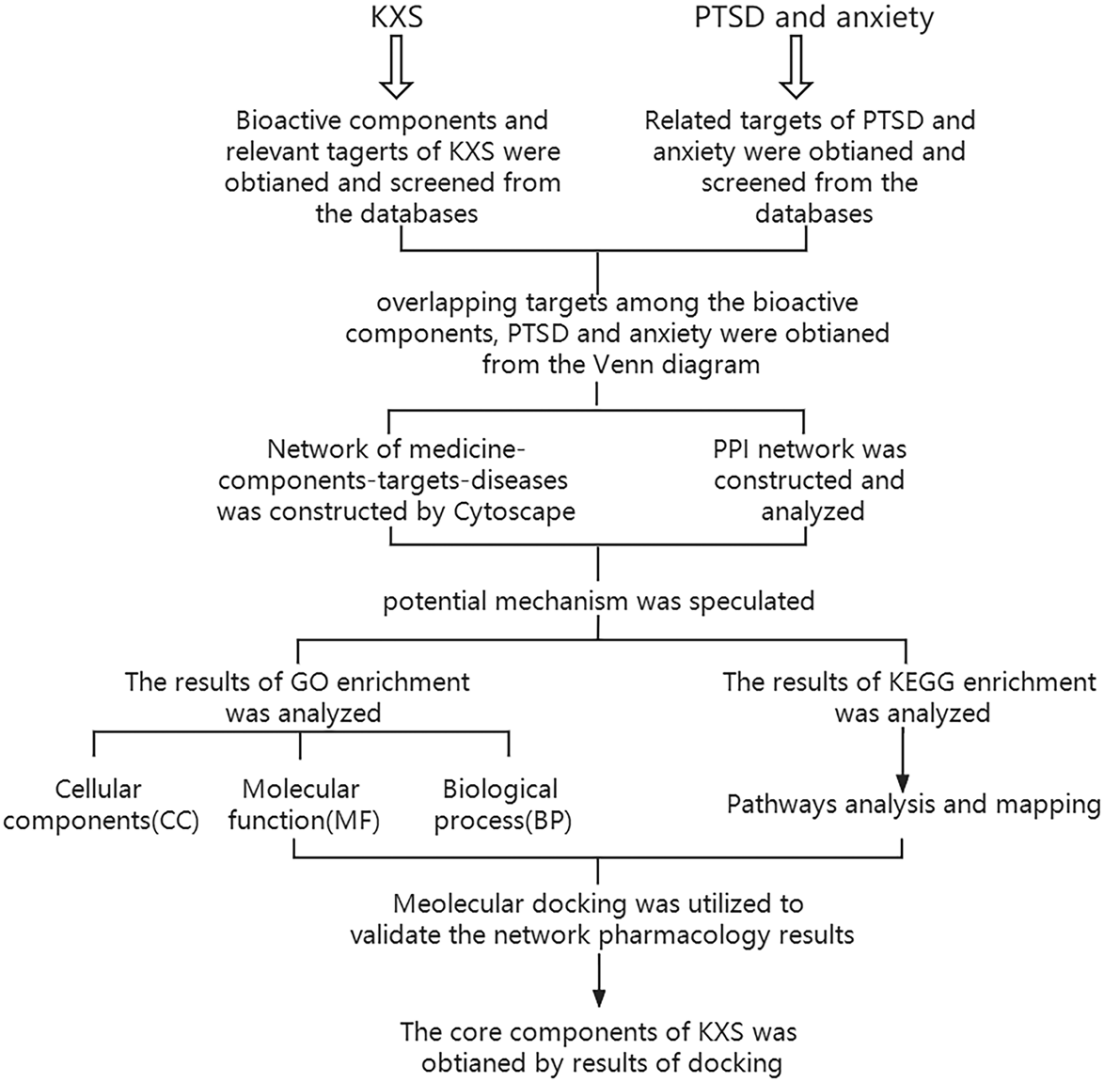
Schematic diagram of the process of network pharmacology analysis with molecular docking.

Due to the lower side effect and efficacy of treating mental disorders of KXS, the method of network pharmacology study was used to collect the bioactive components and explore the therapeutic mechanisms of KXS, and then molecular docking was used to predict potential ligand-protein binding. Herein, this study has 5 phases (see Fig. 1): the active components and relevant targets of KXS were obtained. Targets of PTSD and anxiety were obtained. The visualized networks were built and analyzed. The key components, targets, and correlated signal pathways were found and analyzed. The most valuable components were obtained.

## 2. Materials and methods

### 2.1. Collecting and screening the active components and relevant targets of KXS

The bioactive components and relevant targets of KXS were obtained from the following databases: Traditional Chinese Medicine Systems Pharmacology Database and Analysis Platform (TCMSP) database (https://tcmspw.com/tcmsp.php), Bioinformatics Analysis Tool for Molecular mechanism of Traditional Chinese Medicine (http://bionet.ncpsb.org/batman-tcm/) and Swiss-ADME (http://www.swissadme.ch/).^[14,15]^ In the TCMSP database, the bioactive components were screened and obtained by setting TCMSP suggested drug screening criteria: molecular weight ≤ 500, oral bioavailability ≥ 20%, blood–brain barrier ≥ −0.3, and drug-likeness ≥ 0.10. And then, the Bioinformatics Analysis Tool for Molecular mechanism of Traditional Chinese Medicine was used to obtain bioactive components and screened by Swiss-ADME. In Swiss-ADME, The bioactive components were selected by the results of “High” gastrointestinal absorption and at least two of the 5 drug-likeness principles including Lipinski, Ghost, Veber, Egan, and Muege.^[16,17]^ The relevant targets were collected after screening bioactive components and the names of relevant target genes were obtained and verified from the UniProt database.^[13]^

### 2.2. Collecting target genes of PTSD and anxiety

The protein of “Homo sapiens” was selected. The targets of PTSD and anxiety were obtained from the database, including the DrugBank database (https://www.drugbank.ca/), GeneCards (https://www.genecards.org/), Online Mendelian Inheritance in Man (https://www.omim.org/), DisGeNet database (https://www.disgenet.org/), and Therapeutic Targets Database (https://db.idrblab.net/ttd/).^[18,19]^ The target gene names were also verified by the UniProt database (https://www.uniprot.org/).

### 2.3. Collecting the overlapped genes between bioactive components and diseases

The Venny 2.1.0 (https://bioinfogp.cnb.csic.es/tools/venny/) was used to obtain the overlapping genes among bioactive components, anxiety, and PTSD.^[20]^ The overlapping genes were used to predict the potential molecular mechanisms of KXS for anxiety and PTSD. After collection, the result was shown as a Venn diagram and the network of medicine-components-targets-diseases was constructed by Cytoscape 3.8.0 software.^[21]^

### 2.4. Constructing and analyzing protein–protein interaction (PPI) network

To explore the interaction of overlapping genes and screen the key proteins, the STRING database (https://string-db.org/) was used to construct the PPI network by the following steps: the overlapping genes were inputted into the STRING database. The species was selected as “Homo. sapiens.” The minimum required interaction score was selected as “0.400.” “hide disconnected nodes in the network” were selected. And then, the PPI network was performed. And then, the result was saved as a tsv file and analyzed by Cytoscape software3.8.0.^[22]^

### 2.5. Gene ontology (GO) and Kyoto encyclopedia of genes and genomes (KEGG) pathway enrichment analysis

In this study, the R package clusterProfiler was used to perform GO and KEGG enrichment analysis by setting “H. sapiens” and threshold value as “*P* < .05.” GO and KEGG enrichment analysis are computational approaches that can provide molecular function (MF), cellular components (CC), biological process (BP), and signaling pathways to explore the mechanisms of KXS further. In addition, the counts of genes in signaling pathways or GO were calculated to predict the potential mechanisms.^[13,23]^ Cytoscape 3.8.0 was utilized to construct the networks of KEGG and GO enrichment. Moreover, the mappings of signaling pathways were obtained from the R language.

### 2.6. Molecular docking

Molecular docking is a method to validate the association of components-targets in network pharmacology study.^[24]^ The bioactive components and core target proteins were selected from the network of medicine-components-targets-diseases and PPI analysis, respectively. And then, the molecular structures of the components were saved as mol2 files in the TCMSP database and PubChem database. The 3D structures of target proteins were downloaded and saved as PDB format files from RCSB Protein Data Bank and modified by PyMOL2.6.0, mainly containing the removal of water molecules and organics. In Auto-docking 4.2.6 software, the ligands and proteins were pretreated to perform the docking process by using the genetic algorithm docking protocol. The grid boxes which covered the entire protein were constructed to perform blind docking. All docking parameters were set by default for the algorithm. Binding energy represents the docking score, and the lower binding energy represents the most possible dominant conformation. The binding energy of protein-ligands was compared with the binding energy of positive drugs for target proteins. The docking of protein-ligands with the lowest binding energy was deemed as the target of binding with the most possibilities.^[25,26]^ The docking results were visualized by PyMOL2.6.0 software. Protein-ligand complexes and their position of amino acid residues were evaluated.^[27]^

## 3. Results

### 3.1. Potential targets of KXS and diseases

By screening criteria of TCMSP and Swiss-ADME, 64 active components (i.e., MOL008570, MOL000935, MOL005396, and MOL000066, etc) and 270 target genes (AKT1, IL6, IL8, NOS1, and NOS2, etc) were selected from KXS. The details of bioactive components were shown in Table 1. Moreover, 3474 target genes of PTSD and 4910 target genes of anxiety were selected.

**Table 1:** Bio-active components in KXS.

ID	Molecule name	ID	Molecule name
MOL005321	Frutinone A	MOL000787	Fumarine
MOL005386	Vulgarin	MOL005384	suchilactone
MOL000449	Stigmasterol	MOL000749	Linoleic
MOL000935	Hepanal	MOL002879	Diop
MOL000358	beta-sitosterol	MOL005308	Aposiopolamine
MOL000676	DBP	MOL005356	Girinimbin
MOL003648	Inermin	MOL001312	9-HEXADECENOIC ACID
MOL005272	13-Tetradecenyl acetate	MOL000305	lauric acid
MOL005366	Malvic acid	MOL003576	(1R,3aS,4R,6aS)-1,4-bis(3,4-dimethoxyphenyl)-1,3,3a,4,6,6a-hexahydrofuro[4,3-c]furan
MOL001641	METHYL LINOLEATE	MOL003578	Cycloartenol
MOL001818	Methyl palmitelaidate	MOL001944	Marmesin
MOL005376	Panaxadiol	MOL002955	2’-O-Methylisoliquiritigenin
MOL005399	alexandrin_qt	MOL003553	Calamendiol
MOL005352	Ginsenoyne B	MOL003543	Aminacrin
MOL005351	Ginsenoyne A	MOL003561	isopimpinellin
MOL005355	Ginsenoyne E	MOL003560	Isocalamendiol
MOL000275	trametenolic acid	MOL003577	Bisasaricin
MOL000282	ergosta-7,22E-dien-3beta-ol	MOL003566	Murolan-3,9(11)-diene-10-peroxy
MOL000283	Ergosterol peroxide	MOL000333	Veraguensin
MOL000296	hederagenin	MOL003546	Aristolone
MOL000301	2-lauroleic acid	MOL003571	spathulenol
MOL000302	Undekansaeure	MOL003541	()-alpha-Longipinene
MOL005285	20(s)-protopanaxadiol	MOL003568	Patchoulene
MOL000066	alloaromadedrene	MOL001566	calarene
MOL005396	cis-Widdrol alpha-epoxide	MOL003575	α-Panasinsene
MOL005269	(+)-Maalioxide	MOL000266	beta-Cubebene
MOL004100	N-Salicylidene-salicylamine	MOL003538	()-Ledene
MOL005320	arachidonate	MOL002140	Perlolyrine
MOL005348	Ginsenoside-Rh4_qt	MOL008570	Harmine
MOL001179	(−)-Alloaromadendrene	MOL002003	(-)-Caryophyllene oxide
MOL000612	(-)-alpha-cedrene	N\A1	Tenulin
MOL002335	beta-Gurjunene	N\A2	S-(2-Carboxyethyl)-L-Cysteine

KXS = Kaixinsan powder.

### 3.2. The intersection of potential targets

**Figure 2.**
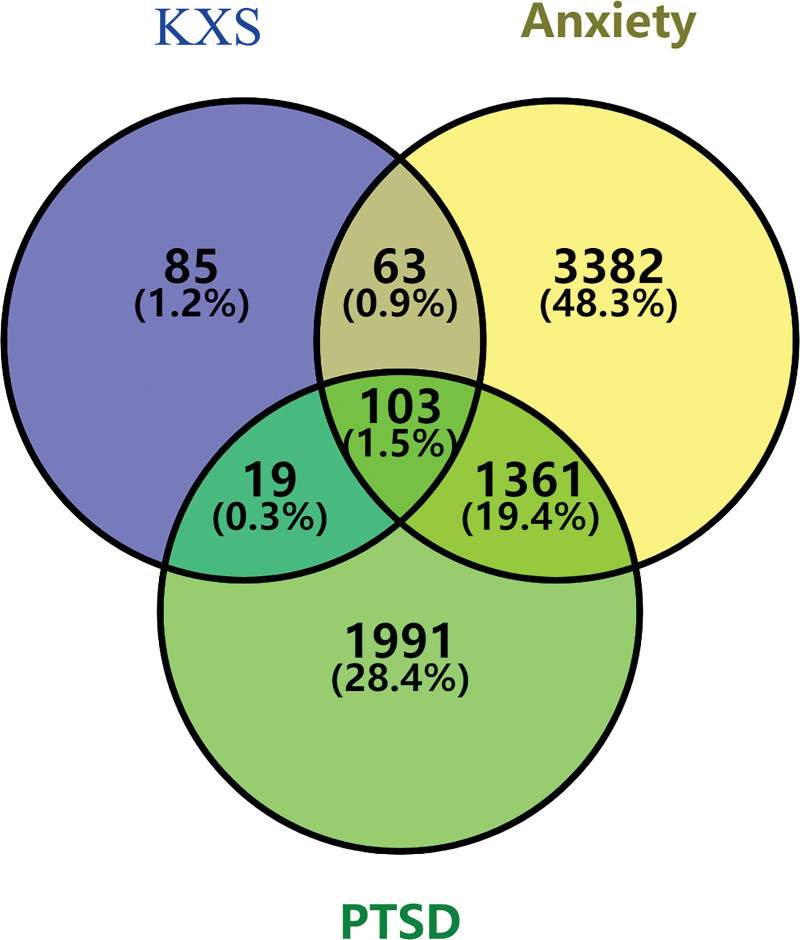
Venn diagram showed the overlapping gene among PTSD, anxiety and KXS. KXS = Kaixinsan powder, PTSD = Post-traumatic stress disorder.

As shown in Figure 2, 103 overlapping genes were derived via Venn diagram, including ABAT, ACHE, ADNP, ADRA1A, ADRA1B, ADRA1D, ADRA2A, ADRA2B, ADRA2C, ADRB1, ADRB2, AKT1, APOD, ARG1, BCHE, BCL2, CA2, CALM1, CAMK2G, CASP3, CASP8, CAV1, CCNA2, CD80, CD86, CHRM1, CHRM2, CHRM3, CHRM4, CHRM5, CHRNA2, CHRNA7, COMT, CPQ, CYP19A1, CYP3A4, DPP4, DRD1, DRD2, EDN1, ESR1, ESR2, F10, F2, F7, FLT3, GABRA1, GABRA2, GABRA3, GABRA5, GABRA6, GCLC, GLRA2, GLUL, GPT, GPT2, GRIA2, GRIN2A, GSK3B, HTR2A, HTR3A, IL6, JUN, KCNH2, KDR, MAOA, MAOB, MAP2, MAPK14, MMP2, MTR, NOS1, NOS2, NOS3, NR3C1, NR3C2, OPRD1, OPRM1, OTC, PDE2A, PDE4A, PDE4D, PDE5A, PLAU, PON1, PPARG, PRKACA, PRKCA, PTGS1, PTGS2, RELA, SCN5A, SHMT2, SLC1A1, SLC1A4, SLC1A5, SLC6A2, SLC6A3, SLC6A4, SLC6A9, SLC7A1, TGFB1, and VDAC1. Overlapping genes accounted for 1.5% in total.

### 3.3. Network of medicine-components-targets-disease construction

**Figure 3.**
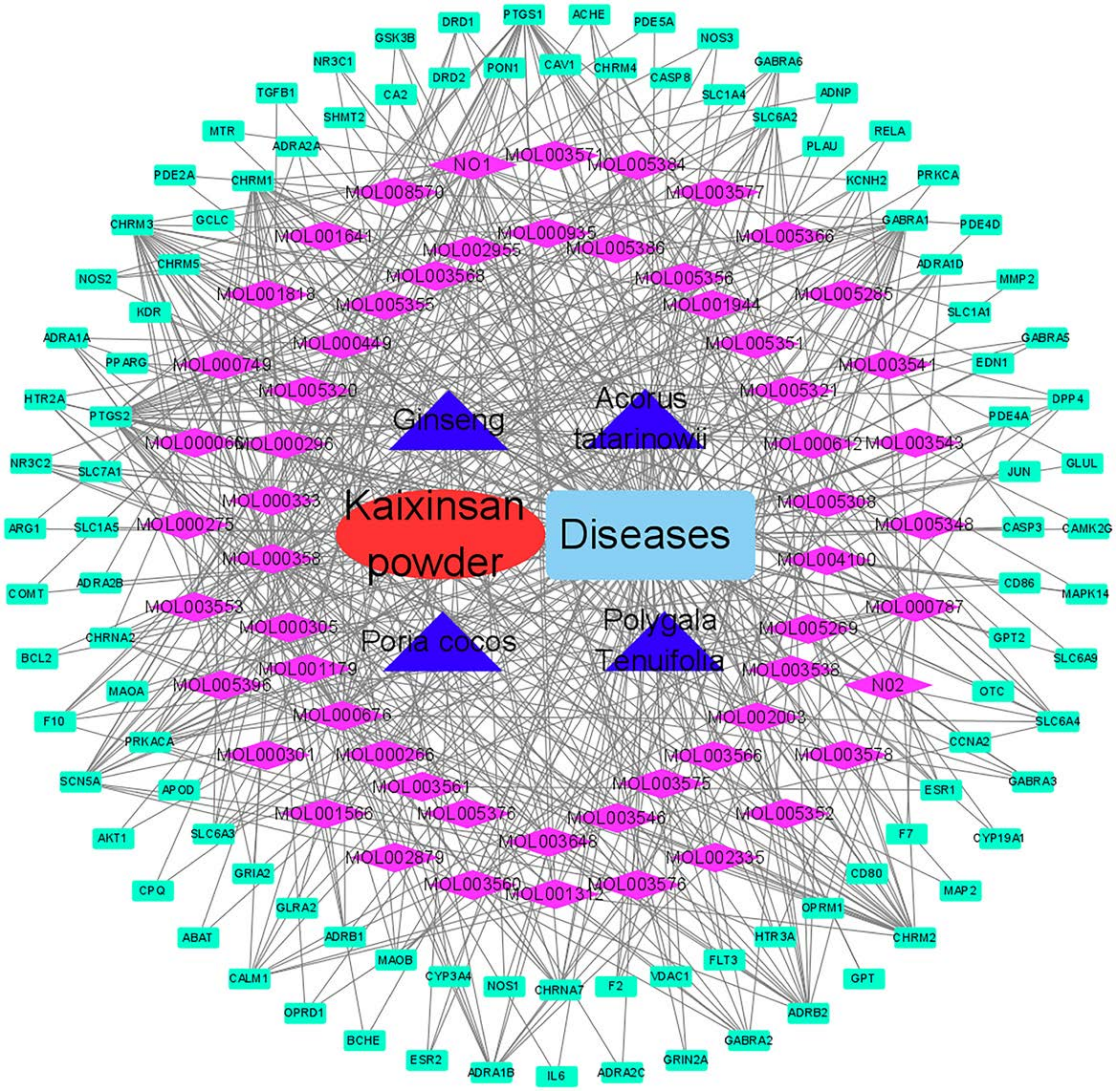
The medicine-components-overlapping targets-diseases network construction. The network included 167 nodes and 577 edges. Green rectangles represented overlapping targets, purple rhombus represented bioactive components, blue triangles represented medicine, red oval represented KXS and Wathet rectangle represented diseases. KXS = Kaixinsan powder.

The network construction of medicine-components-targets-disease is shown in Figure 3 and Table 2. There are 58 bioactive components and 103 overlapping genes with 167 nodes and 577 edges. Moreover, the Cytoscape 3.8.0 software was used to analyze other parameters, like network density (0.021), Characteristic path length (1.586), and average number of neighbors (6.910). The target gene with the maximum degree was PTGS2 (32) and the component with the maximum degree was beta-sitosterol (31). And then, the components with the top 4 degrees in the network were beta-sitosterol (31), Tenulin (28), Fumarine (25), and Stigmasterol (23), respectively. The network predicted that the multiple bioactive components and target genes mediated the same pathogeny of anxiety and PTSD.

**Table 2:** Target genes interacting with components in the medicine-components-overlapping targets-diseases network.

ID	Molecule name	Gene name
MOL000066	alloaromadedrene	CHRM1/CHRM2/CHRM3/CHRNA2/GABRA1
MOL000266	beta-Cubebene	SLC6A2/PTGS2/GABRA1/CHRNA2/CHRM2/CHRM1
MOL000275	trametenolic acid	NR3C2
MOL000296	hederagenin	ADRA1B/CHRM1/CHRM2/CHRM3/GABRA1/GABRA2/GABRA3/GABRA5/GABRA6/GRIA2/PTGS1/PTGS2/SCN5A/SLC6A2
MOL000301	2-lauroleic acid	PTGS1
MOL000305	lauric acid	AKT1/BCHE/CD80/CD86/IL6/PTGS1/PTGS2/RELA
MOL000333	Veraguensin	SLC6A3/SCN5A/PTGS2/PTGS1/OPRD1/KCNH2/F10/ESR1/DRD2/DRD1/CHRM5/CHRM4/CHRM3/CHRM1/CALM1/ADRB2/ADRA2C/ADRA1D/ADRA1B/ADRA1A/ACHE
MOL000358	beta-sitosterol	ADRA1A/ADRA1B/ADRB2/BCL2/CASP3/CASP8/CHRM1/CHRM2/CHRM3/CHRM4/CHRNA2/CHRNA7/DRD1/GABRA1/GABRA2/GABRA3/GABRA5/HTR2A/JUN/KCNH2/MAP2/OPRM1/PON1/PRKACA/PRKCA/PTGS1/PTGS2/SCN5A/SLC6A4/TGFB1
MOL000449	Stigmasterol	ADRA1A/ADRA1B/ADRA2A/ADRB1/ADRB2/CHRM1/CHRM2/CHRM3/CHRNA7/GABRA1/GABRA3/HTR2A/MAOA/MAOB/NR3C2/PLAU/PRKACA/PTGS1/PTGS2/SCN5A/SLC6A2/SLC6A3
MOL000612	(-)-alpha-cedrene	PTGS2/GABRA2/GABRA1/CHRM3/CHRM1
MOL000676	DBP	ADRA1A/ADRB2/CHRM1/CHRM2/CHRM3/CHRNA7/GABRA1/GABRA6/GSK3B/HTR2A/NOS3/PRKACA/PTGS1/PTGS2/SLC6A2/SLC6A3/SLC6A4
MOL000749	Linoleic	ADRA2A/ADRA2B/ADRB1/ADRB2/CHRM1/CHRM2/MAOB/PTGS1/PTGS2/SLC6A2/SLC6A3/SLC6A4
MOL000787	Fumarine	ADRA1B/ADRA1D/ADRB2/CALM1/CHRM1/CHRM3/CHRM4/CHRM5/DRD1/F10/F7/HTR2A/HTR3A/KCNH2/KDR/OPRD1/OPRM1/PDE4A/PRKACA/PTGS1/PTGS2/SCN5A/SLC6A3/SLC6A4
MOL000935	Hepanal	CHRM2/CHRM3
MOL001179	(-)-Alloaromadendrene	SLC6A3/SLC6A2/HTR2A/GABRA3/GABRA1/DRD1/CHRNA7/CHRNA2/CHRM3/CHRM2/CHRM1/ADRB2/ADRB1/ADRA1B/ADRA1A
MOL001312	9-HEXADECENOIC ACID	CALM1/GABRA1/GABRA2/PRKACA/PTGS1/PTGS2/SLC6A2
MOL001566	calarene	CHRM3
MOL001641	METHYL LINOLEATE	PTGS1/PTGS2
MOL001818	Methyl palmitelaidate	PTGS1/PTGS2
MOL001944	Marmesin	SLC6A4/PTGS2/PTGS1/PRKACA/F2/ESR1/DPP4/CHRM2/CHRM1/ADRB2
MOL002003	(-)-Caryophyllene oxide	PTGS2/GABRA6/GABRA1/F2/DPP4/CHRM3/CHRM2/CHRM1/ADRA1B/ACHE
MOL002335	beta-Gurjunene	GABRA1/CHRNA2/CHRM3/CHRM2/CHRM1/ADRA1B
MOL002879	Diop	ADRB2/CHRM3/SCN5A
MOL002955	2’-O-Methylisoliquiritigenin	SLC6A4/PTGS2/PTGS1/PRKACA/PPARG/NOS2/MAPK14/MAOB/GSK3B/ESR2/ESR1/CCNA2/CALM1/CA2/ADRB2
MOL003538	()-Ledene	CHRM3/CHRM1
MOL003541	()-alpha-Longipinene	GABRA1/CHRNA2/CHRM3/CHRM2
MOL003543	Aminacrin	SCN5A/PTGS2/PTGS1/PRKACA/MAOB
MOL003546	Aristolone	PTGS2/GABRA1/CHRM3/CHRM2/CHRM1
MOL003553	Calamendiol	PTGS2/GRIA2/GABRA6/GABRA2/GABRA1/CHRNA7/CHRM3/CHRM2/CHRM1
MOL003560	Isocalamendiol	GABRA6/GABRA2/GABRA1/CHRM2/CHRM1
MOL003561	isopimpinellin	PDE4A/ESR1/CYP3A4
MOL003566	Murolan-3/9(11)-diene-10-peroxy	GABRA6/GABRA1/CHRM3/CHRM2/CHRM1
MOL003568	Patchoulene	GABRA2/GABRA1/CHRM3/CHRM2/CHRM1
MOL003571	spathulenol	CHRM3/CHRM2/CHRM1
MOL003575	α-Panasinsene	CHRM3/CHRM2
MOL003576	(1R/3aS/4R/6aS)-1/4-bis(3/4-dimethoxyphenyl)-1/3/3a/4/6/6a-hexahydrofuro[4/3-c]furan	SCN5A/PTGS2/KCNH2/F10/CHRM3/CALM1/ADRA1D/ADRA1B
MOL003577	Bisasaricin	SCN5A/PTGS2/NOS2/F10/ESR2/ESR1/CALM1
MOL003578	Cycloartenol	NR3C2
MOL003648	Inermin	ADRA1B/ADRA1D/ADRB2/CALM1/CHRM3/CHRNA7/HTR3A/PRKACA/PTGS1/PTGS2/SCN5A/SLC6A4
MOL004100	N-Salicylidene-salicylamine	DPP4/MAOB/MAPK14/PRKACA/PTGS1/PTGS2
MOL005269	(+)-Maalioxide	CHRM2/CHRM3
MOL005285	20(s)-protopanaxadiol	CASP3/MMP2
MOL005308	Aposiopolamine	ADRB2/CHRM1/CHRM3/DPP4/GABRA1/SLC6A2/SLC6A3/SLC6A4
MOL005320	arachidonate	PTGS1/PTGS2
MOL005321	Frutinone A	ACHE/ADRB2/CHRNA7/DPP4/F2/GABRA1/PPARG/PRKACA/PTGS1/PTGS2/SCN5A
MOL005348	Ginsenoside-Rh4_qt	NR3C2
MOL005351	Ginsenoyne A	PTGS2
MOL005352	Ginsenoyne B	PTGS1/PTGS2
MOL005355	Ginsenoyne E	PTGS2
MOL005356	Girinimbin	ADRB2/CHRNA7/GABRA1/PRKACA/PTGS1/PTGS2/SCN5A
MOL005366	Malvic acid	PTGS1/PTGS2/SLC6A2
MOL005376	Panaxadiol	NR3C1
MOL005384	suchilactone	ADRA1D/ADRB2/CALM1/F10/F7/KCNH2/PRKACA/PTGS1/PTGS2/SCN5A
MOL005386	Vulgarin	ACHE/GABRA1
MOL005396	cis-Widdrol alpha-epoxide	CHRM1/CHRM2/CHRM3/GABRA1
MOL008570	Harmine	CAMK2G/PDE4D/PDE5A/PDE2A
N\A1	Tenulin	NR3C1/NR3C2/CYP19A1
N\A2	S-(2-Carboxyethyl)-L-Cysteine	ARG1/CPQ/VDAC1/OTC/EDN1/NOS2/CAV1/GCLC/SLC1A5/GLRA2/GRIN2A/GPT/SLC6A9/GLUL/ADNP/NOS3/MTR/COMT/ABAT/FLT3/GPT2/SLC1A1/SLC1A4/SHMT2/SLC7A1/APOD/NOS1

### 3.4. Network of PPI

**Figure 4.**
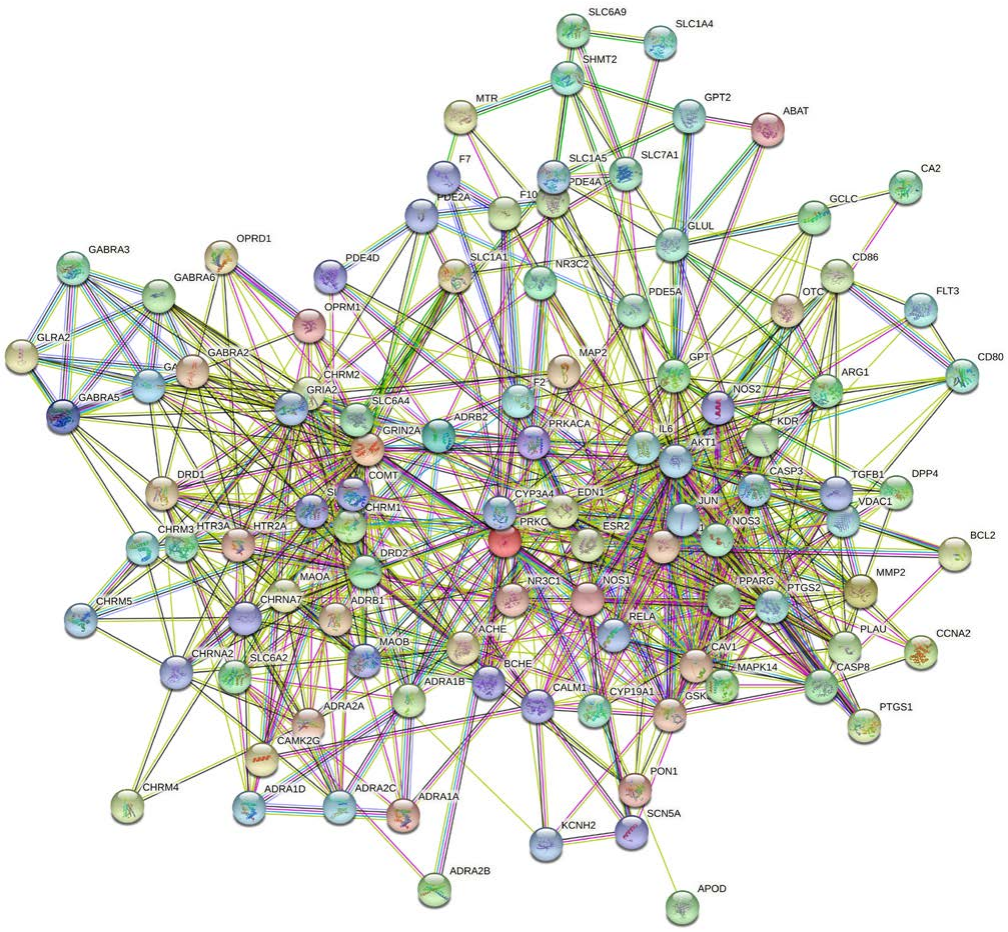
Network of PPI analysis. PPI network analysis included the interaction of 103 overlapping protein. PPI = protein–protein interaction.

As shown in Figure 4, 103 overlapping proteins were utilized to construct the network of PPI, including ABAT, ACHE, ADNP, ADRA1A, ADRA1B, ADRA1D, ADRA2A, ADRA2B, ADRA2C, ADRB1, ADRB2, AKT1, APOD, ARG1, BCHE, BCL2, CA2, CALM1, CAMK2G, CASP3, CASP8, CAV1, CCNA2, CD80, CD86, CHRM1, CHRM2, CHRM3, CHRM4, CHRM5, CHRNA2, CHRNA7, COMT, CPQ, CYP19A1, CYP3A4, DPP4, DRD1, DRD2, EDN1, ESR1, ESR2, F10, F2, F7, FLT3, GABRA1, GABRA2, GABRA3, GABRA5, GABRA6, GCLC, GLRA2, GLUL, GPT, GPT2, GRIA2, GRIN2A, GSK3B, HTR2A, HTR3A, IL6, JUN, KCNH2, KDR, MAOA, MAOB, MAP2, MAPK14, MMP2, MTR, NOS1, NOS2, NOS3, NR3C1, NR3C2, OPRD1, OPRM1, OTC, PDE2A, PDE4A, PDE4D, PDE5A, PLAU, PON1, PPARG, PRKACA, PRKCA, PTGS1, PTGS2, RELA, SCN5A, SHMT2, SLC1A1, SLC1A4, SLC1A5, SLC6A2, SLC6A3, SLC6A4, SLC6A9, SLC7A1, TGFB1, and VDAC1.

**Figure 5.**
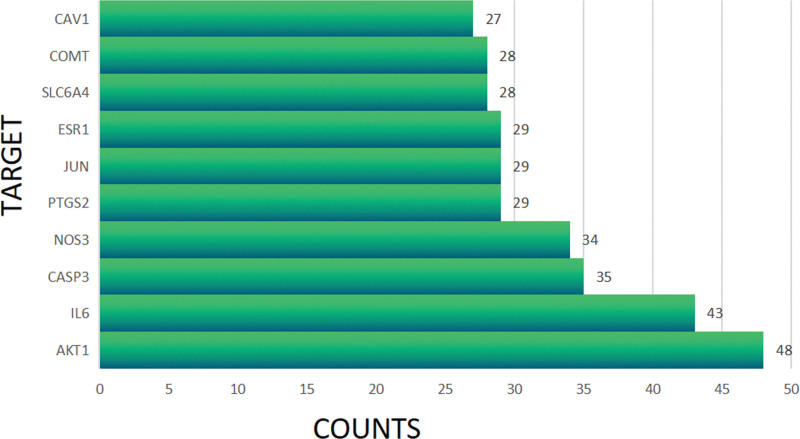
Proteins with top 10 node degree. The top 10 proteins in terms of node degree included AKT1, IL-6, CASP3, NOS3, PTGS2, JUN, ESR1, SLC6A4, COMT, and CAV1.

The results were analyzed and processed by cytoscape3.8.0 software: number of nodes (103) and edges (693), average node degree (13.5), and average local clustering coefficient (0.513). After, the degree of each node was shown in Figure 5. The proteins with the top 2 degrees were AKT1 and IL6. The results indicated that AKT1 and IL6 may play important role in the network of PPI.

### 3.5. GO enrichment analysis of target genes

**Figure 6.**
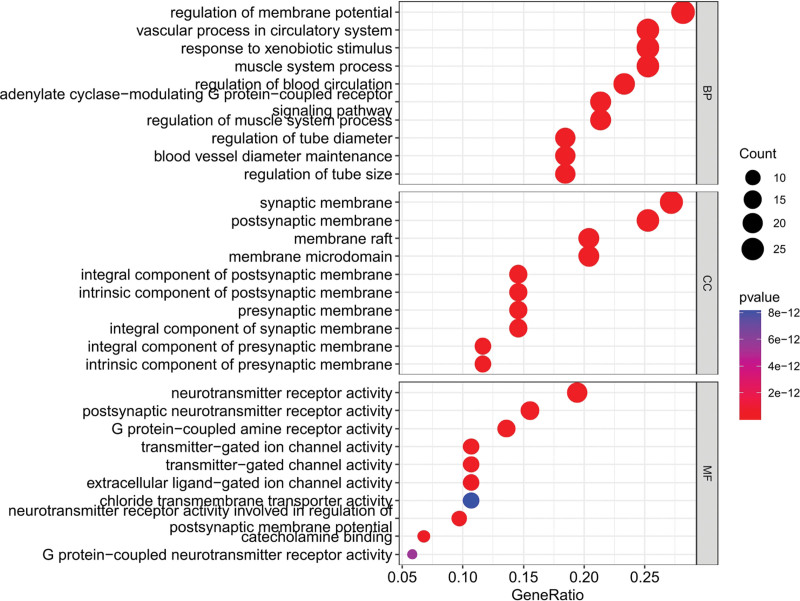
The top 10 gene-ratio term in BP, CC, and MF. The *y* axis showed the BP, CC, and MF respectively. The *x* axis showed the enrichment scores of these term, and the size of the circles mean that counts of gene-ratio of each term. BP = biological process, CC = cellular components, MF = molecular function.

**Figure 7.**
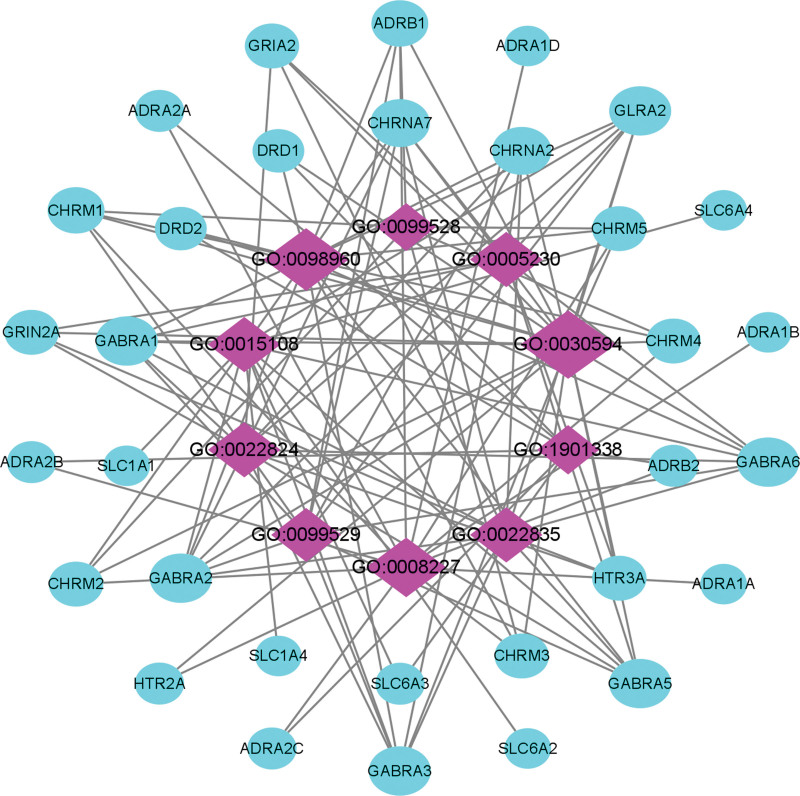
Network construction of MF in GO enrichment analysis. The network consisted of 42 nodes and 117 edges. Blue circles represented targets gene, and purple rhombus represented core MF. GO = gene ontology, MF = molecular function.

GO enrichment analysis is employed to speculate the function of overlapping genes from MF, CC, and BP. By setting the threshold value of p < .05, the top 3 (gene-ratio) terms of GO enrichment analysis had been obtained. The results of MF in the top 3 terms were neurotransmitter receptor activity, postsynaptic neurotransmitter receptor activity, and G protein-coupled amine receptor activity (Fig. 6, Table 3). Topological properties of the network construction of MF were shown that the number of nodes and edges was 42 and 117, respectively (Fig. 7). Moreover, we also analyzed other parameters, such as the average number of neighbors (5.571) and network density (0.068). The MF and target genes with maximum degree were neurotransmitter receptor activity (20) and GLRA2/GABRA6/GABRA5/GABRA3/GABRA2/GABRA1 (7) respectively.

**Table 3:** Molecular function in GO enrichment analysis.

ID	Molecular function	*P* value	Count	Gene name
GO:0030594	neurotransmitter receptor activity	4.59E-25	20	ADRB1/CHRM1/CHRM2/CHRM3/CHRM4/CHRM5/CHRNA2/CHRNA7/DRD1/DRD2/GABRA1/GABRA2/GABRA3/GABRA5/GABRA6/GLRA2/GRIA2/GRIN2A/HTR2A/HTR3A
GO:0098960	postsynaptic neurotransmitter receptor activity	1.07E-22	16	ADRB1/CHRM1/CHRM2/CHRM3/CHRM4/CHRM5/CHRNA2/CHRNA7/DRD1/DRD2/GABRA1/GABRA2/GABRA3/GABRA5/GABRA6/GLRA2
GO:0008227	G protein-coupled amine receptor activity	1.28E-20	14	ADRA1A/ADRA1B/ADRA1D/ADRA2A/ADRA2B/ADRA2C/ADRB1/ADRB2/CHRM1/CHRM2/CHRM3/CHRM4/CHRM5/HTR2A
GO:0022824	transmitter-gated ion channel activity	2.65E-14	11	CHRNA2/CHRNA7/GABRA1/GABRA2/GABRA3/GABRA5/GABRA6/GLRA2/GRIA2/GRIN2A/HTR3A
GO:0022835	transmitter-gated channel activity	2.65E-14	11	CHRNA2/CHRNA7/GABRA1/GABRA2/GABRA3/GABRA5/GABRA6/GLRA2/GRIA2/GRIN2A/HTR3A
GO:0005230	extracellular ligand-gated ion channel activity	2.60E-13	11	CHRNA2/CHRNA7/GABRA1/GABRA2/GABRA3/GABRA5/GABRA6/GLRA2/GRIA2/GRIN2A/HTR3A
GO:0015108	chloride transmembrane transporter activity	8.16E-12	11	GABRA1/GABRA2/GABRA3/GABRA5/GABRA6/GLRA2/SLC1A1/SLC1A4/SLC6A2/SLC6A3/SLC6A4
GO:0099529	neurotransmitter receptor activity involved in regulation of postsynaptic membrane potential	1.32E-13	10	ADRB1/CHRM1/CHRNA2/CHRNA7/GABRA1/GABRA2/GABRA3/GABRA5/GABRA6/GLRA2
GO:1901338	catecholamine binding	2.33E-13	7	ADRA2A/ADRA2B/ADRA2C/ADRB2/DRD1/DRD2/SLC6A3
GO:0099528	G protein-coupled neurotransmitter receptor activity	5.46E-12	6	ADRB1/CHRM1/CHRM2/CHRM3/CHRM4/CHRM5

GO = gene ontology.

**Figure 8.**
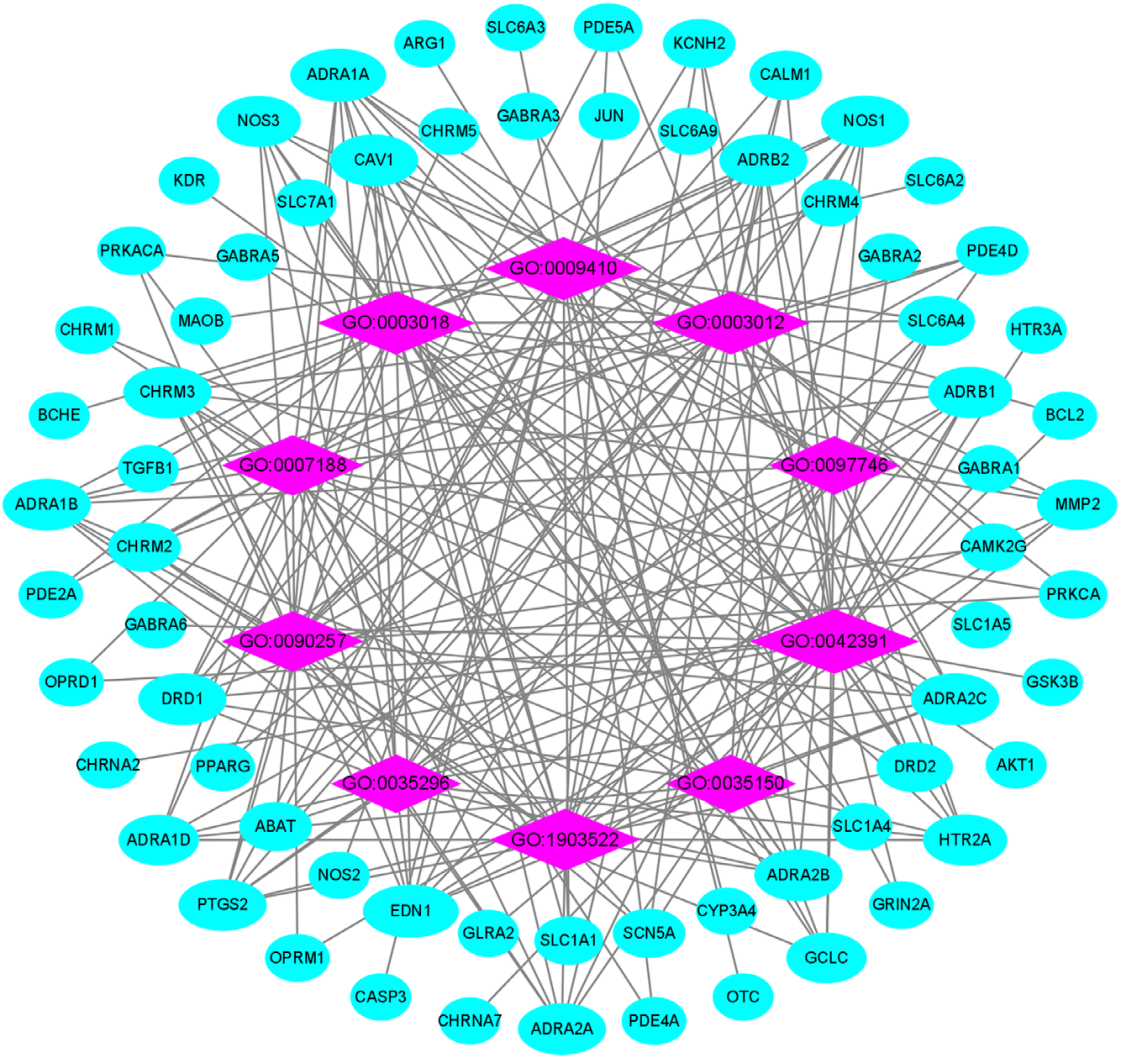
Network construction of BP in GO enrichment analysis. The network consisted of 78 nodes and 232 edges. Blue circles represented targets gene, and purple rhombus represented core BP. BP = biological process, GO = gene ontology.

The top 3 (gene-ratio) terms of BP were regulation of membrane potential, vascular process in the circulatory system, and response to xenobiotic stimulus (Fig. 6, Table 4). Topological properties of the network construction of BP were shown that the number of nodes and edges was 78 and 232, respectively (Fig. 8). Moreover, other parameters also were analyzed, such as average number of neighbors (5.949) and network density (0.039). The BP and target genes with maximum degree were regulation of membrane potential (29) and ADRA1A/EDN1 (10), respectively.

**Table 4:** Biological process in GO enrichment analysis.

ID	Biological process	*P* value	Count	Gene name
GO:0042391	regulation of membrane potential	5.30E-24	29	ABAT/ADRA1A/ADRB1/ADRB2/AKT1/BCL2/CALM1/CAV1/CHRM1/CHRNA2/CHRNA7/DRD1/DRD2/EDN1/GABRA1/GABRA2/GABRA3/GABRA5/GABRA6/GCLC/GLRA2/GRIN2A/GSK3B/HTR3A/KCNH2/KDR/OPRD1/OPRM1/SCN5A
GO:0003018	vascular process in circulatory system	1.15E-25	26	ADRA1A/ADRA1B/ADRA1D/ADRA2A/ADRA2B/ADRA2C/ADRB1/ADRB2/CAV1/CHRM3/DRD1/EDN1/GCLC/HTR2A/MMP2/NOS1/NOS3/PDE2A/PTGS2/SLC1A1/SLC1A4/SLC1A5/SLC6A4/SLC6A9/SLC7A1/TGFB1
GO:0009410	response to xenobiotic stimulus	1.12E-20	26	ABAT/ADRA1A/ARG1/BCHE/BCL2/CASP3/CYP3A4/DRD1/DRD2/EDN1/GCLC/GRIN2A/HTR2A/JUN/KCNH2/MAOB/MMP2/NOS1/NOS2/OTC/PDE4A/PTGS2/SLC1A1/SLC6A2/SLC6A3/SLC6A4
GO:0003012	muscle system process	1.03E-19	26	ABAT/ADRA1A/ADRA1B/ADRA2A/ADRA2B/ADRA2C/ADRB2/CALM1/CAMK2G/CAV1/CHRM2/CHRM3/DRD1/DRD2/EDN1/HTR2A/KCNH2/NOS1/NOS3/PDE4D/PDE5A/PPARG/PRKACA/PRKCA/PTGS2/SCN5A
GO:1903522	regulation of blood circulation	5.56E-23	24	ADRA1A/ADRA1B/ADRA1D/ADRA2A/ADRA2B/ADRA2C/ADRB1/CALM1/CAV1/CHRM2/CHRM3/DRD2/EDN1/HTR2A/KCNH2/MMP2/NOS1/NOS3/PDE4D/PDE5A/PRKACA/PTGS2/SCN5A/SLC1A1
GO:0007188	adenylate cyclase-modulating G protein-coupled receptor signaling pathway	7.14E-21	22	ADRA1A/ADRA1B/ADRA1D/ADRA2A/ADRA2B/ADRA2C/ADRB1/ADRB2/CHRM1/CHRM2/CHRM3/CHRM4/CHRM5/DRD1/DRD2/EDN1/OPRD1/OPRM1/PDE2A/PDE4A/PDE4D/PRKCA
GO:0090257	regulation of muscle system process	1.22E-20	22	ABAT/ADRA1A/ADRA1B/ADRA2A/ADRA2B/ADRA2C/ADRB2/CALM1/CAMK2G/CAV1/CHRM2/CHRM3/EDN1/NOS1/NOS3/PDE4D/PDE5A/PPARG/PRKACA/PRKCA/PTGS2/SCN5A
GO:0035296	regulation of tube diameter	1.75E-21	19	ADRA1A/ADRA1B/ADRA1D/ADRA2A/ADRA2B/ADRA2C/ADRB1/ADRB2/CAV1/CHRM3/DRD1/EDN1/GCLC/HTR2A/MMP2/NOS1/NOS3/PTGS2/SLC6A4
GO:0097746	blood vessel diameter maintenance	1.75E-21	19	ADRA1A/ADRA1B/ADRA1D/ADRA2A/ADRA2B/ADRA2C/ADRB1/ADRB2/CAV1/CHRM3/DRD1/EDN1/GCLC/HTR2A/MMP2/NOS1/NOS3/PTGS2/SLC6A4
GO:0035150	regulation of tube size	2.01E-21	19	ADRA1A/ADRA1B/ADRA1D/ADRA2A/ADRA2B/ADRA2C/ADRB1/ADRB2/CAV1/CHRM3/DRD1/EDN1/GCLC/HTR2A/MMP2/NOS1/NOS3/PTGS2/SLC6A4

GO = gene ontology.

**Figure 9.**
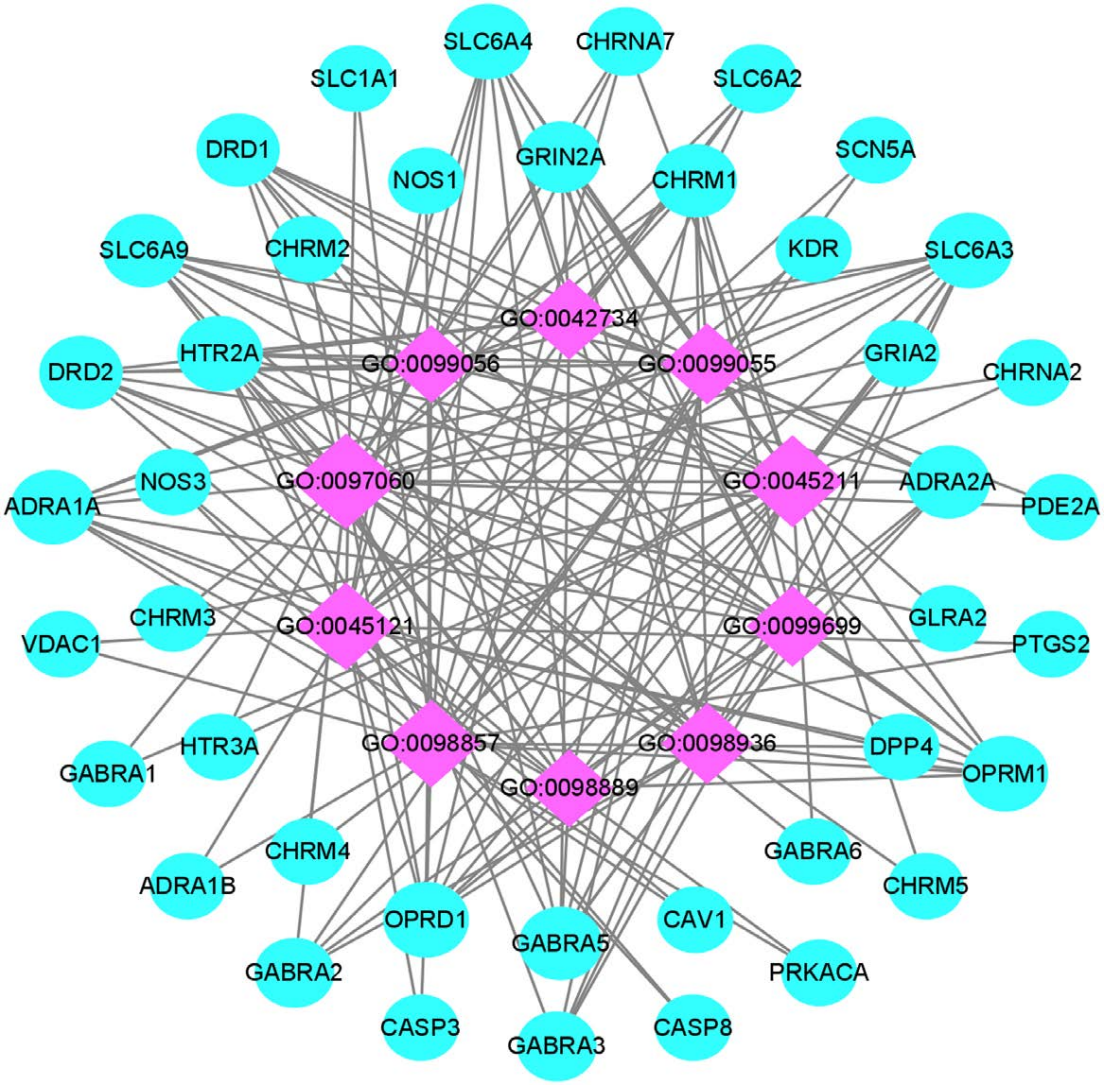
Network construction of CC in GO enrichment analysis. The network consisted of 51 nodes and 179 edges. Blue circles represented targets gene, and purple rhombus represented core CC. CC = cellular components, GO = gene ontology.

The top 3 (gene-ratio) terms of CC were synaptic membrane, postsynaptic membrane, and membrane raft (Fig. 6, Table 5). Topological properties of the network construction of BP show that the number of nodes and edges was 51 and 179, respectively (Fig. 9). Moreover, we also analyzed other parameters, such as the average number of neighbors (7.020) and network density (0.070). The BP and target gene with maximum degree were synaptic membrane (28) and SLC6A4/SLC6A3/OPRM1/OPRD1/HTR2A (10) respectively. The results of GO enrichment analysis indicated that the pharmacological effects of KXS on anxiety and PTSD might be related to neurotransmitter receptor activity, postsynaptic neurotransmitter receptor activity, regulation of membrane potential, vascular process in the circulatory system, synaptic membrane, and postsynaptic membrane.

**Table 5:** Cellular component in GO enrichment analysis.

ID	Cellular component	*P* value	Count	Gene name
GO:0097060	synaptic membrane	8.76E-25	28	ADRA1A/ADRA2A/CHRM1/CHRM2/CHRM3/CHRM4/CHRM5/CHRNA2/CHRNA7/DRD1/DRD2/GABRA1/GABRA2/GABRA3/GABRA5/GABRA6/GLRA2/GRIA2/GRIN2A/HTR2A/HTR3A/OPRD1/OPRM1/PDE2A/SLC6A2/SLC6A3/SLC6A4/SLC6A9
GO:0045211	postsynaptic membrane	8.98E-26	26	ADRA1A/ADRA2A/CHRM1/CHRM2/CHRM3/CHRM4/CHRM5/CHRNA2/CHRNA7/DRD1/DRD2/GABRA1/GABRA2/GABRA3/GABRA5/GABRA6/GLRA2/GRIA2/GRIN2A/HTR2A/HTR3A/OPRD1/OPRM1/SLC6A3/SLC6A4/SLC6A9
GO:0045121	membrane raft	2.74E-17	21	ADRA1A/ADRA1B/CASP3/CASP8/CAV1/CHRNA7/DPP4/HTR2A/KDR/NOS1/NOS3/OPRD1/OPRM1/PRKACA/PTGS2/SCN5A/SLC1A1/SLC6A2/SLC6A3/SLC6A4/VDAC1
GO:0098857	membrane microdomain	2.92E-17	21	ADRA1A/ADRA1B/CASP3/CASP8/CAV1/CHRNA7/DPP4/HTR2A/KDR/NOS1/NOS3/OPRD1/OPRM1/PRKACA/PTGS2/SCN5A/SLC1A1/SLC6A2/SLC6A3/SLC6A4/VDAC1
GO:0099055	integral component of postsynaptic membrane	5.21E-17	15	ADRA1A/ADRA2A/CHRM1/DRD1/DRD2/GABRA2/GABRA3/GABRA5/GRIN2A/HTR2A/OPRD1/OPRM1/SLC6A3/SLC6A4/SLC6A9
GO:0098936	intrinsic component of postsynaptic membrane	9.89E-17	15	ADRA1A/ADRA2A/CHRM1/DRD1/DRD2/GABRA2/GABRA3/GABRA5/GRIN2A/HTR2A/OPRD1/OPRM1/SLC6A3/SLC6A4/SLC6A9
GO:0042734	presynaptic membrane	9.91E-16	15	ADRA1A/ADRA2A/CHRM1/DRD1/DRD2/GABRA5/GRIN2A/HTR2A/OPRD1/OPRM1/PDE2A/SLC6A2/SLC6A3/SLC6A4/SLC6A9
GO:0099699	integral component of synaptic membrane	1.85E-15	15	ADRA1A/ADRA2A/CHRM1/DRD1/DRD2/GABRA2/GABRA3/GABRA5/GRIN2A/HTR2A/OPRD1/OPRM1/SLC6A3/SLC6A4/SLC6A9
GO:0099056	integral component of presynaptic membrane	1.09E-15	12	ADRA1A/ADRA2A/CHRM1/DRD1/DRD2/GABRA5/HTR2A/OPRD1/OPRM1/SLC6A3/SLC6A4/SLC6A9
GO:0098889	intrinsic component of presynaptic membrane	3.86E-15	12	ADRA1A/ADRA2A/CHRM1/DRD1/DRD2/GABRA5/HTR2A/OPRD1/OPRM1/SLC6A3/SLC6A4/SLC6A9

GO = gene ontology.

### 3.6. KEGG enrichment analysis for target genes and mapping

**Figure 10.**
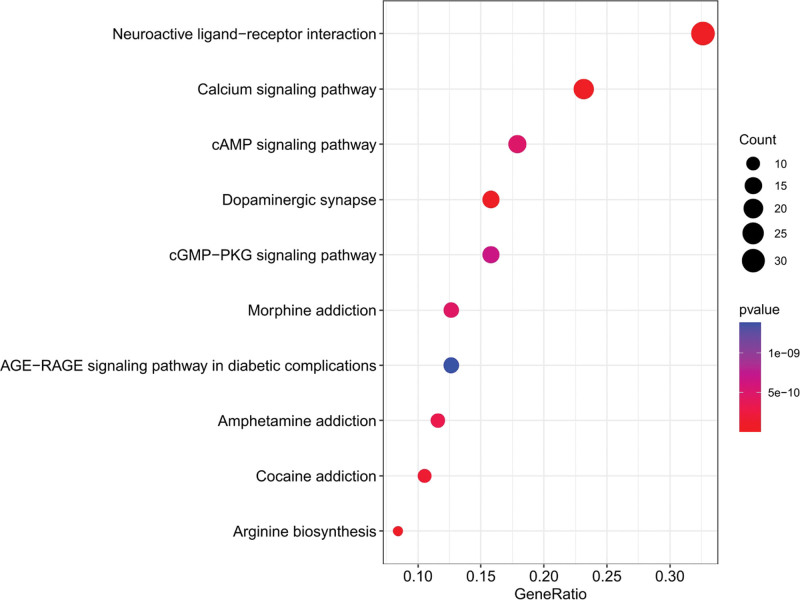
The top 10 gene-ratio term in KEGG enrichment analysis. The *y* axis shown the term of KEGG signaling pathways and the x-axis showed the enrichment scores of these term, and the size of the circles indicated the counts of gene-ratio in each pathway. KEGG = Kyoto encyclopedia of genes and genomes.

**Figure 11.**
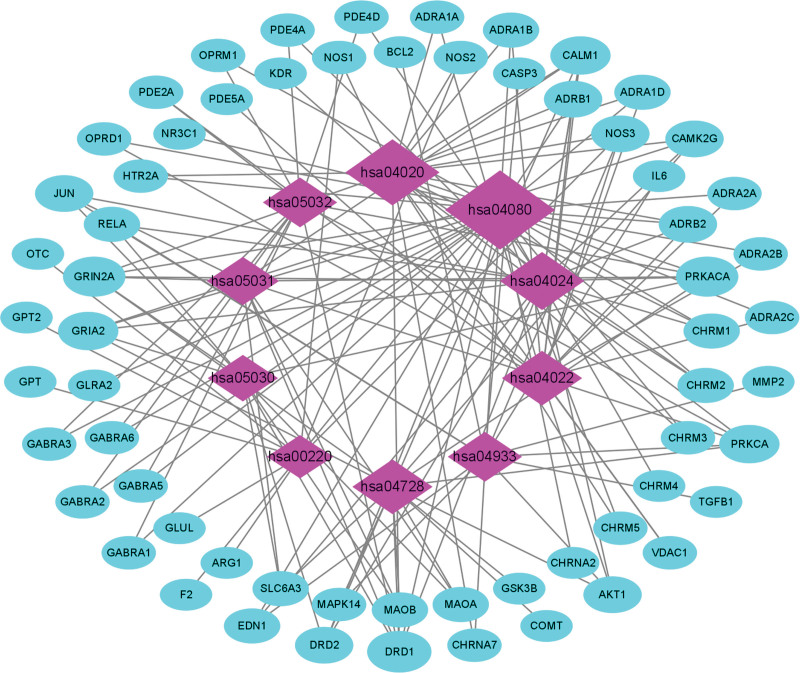
Network construction of KEGG enrichment analysis. The network consisted of 73 nodes and 152 edges. Blue circles represented targets gene, and purple rhombus represented core signaling pathways. KEGG = Kyoto encyclopedia of genes and genomes.

**Figure 12.**
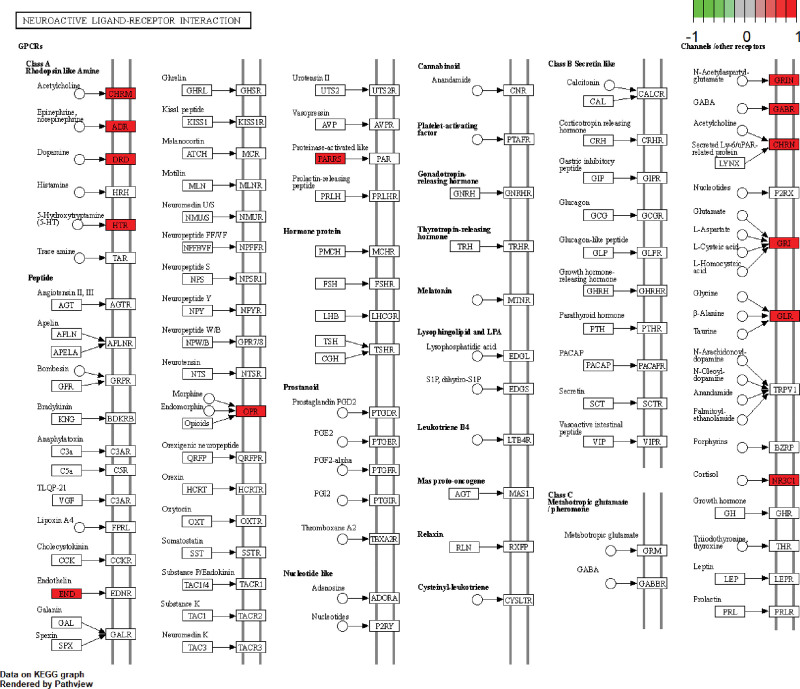
Mapping of neuroactive ligand-receptor interaction.

**Figure 13.**
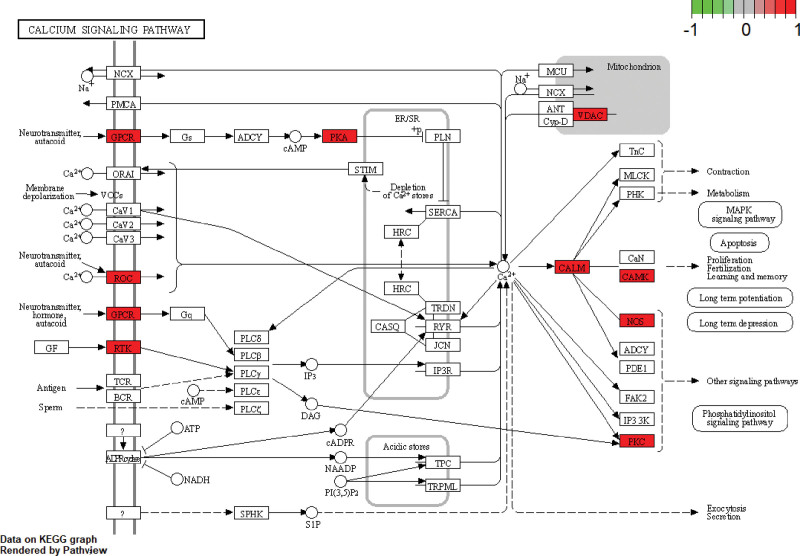
Mapping of calcium signaling pathway.

KEGG enrichment analysis showed that 103 overlapping genes were significantly enriched in 10 signaling pathways (p < .05) (Table 6, Fig. 10). Signaling pathways (gene-ratio) in the top 3 terms were neuroactive ligand-receptor interaction, calcium signaling pathway, and cAMP signaling pathway. The topological properties of KEGG network construction (Fig. 11) showed that the number of nodes and edges was 73 and 152, respectively. Moreover, we also analyzed other parameters, such as average number of neighbors (4.164) and network density (0.029). The signaling pathway and target protein with maximum degree were Neuroactive ligand-receptor interaction (30) and DRD1 (7), respectively. In addition, the mapping of the top 2 signaling pathways was neuroactive ligand-receptor interaction (Fig. 12) and calcium signaling pathway (Fig. 13). KEGG enrichment analysis indicated that neuroactive ligand-receptor interaction and calcium signaling pathway were crucial in the pharmacological effects of bioactive components on PTSD and anxiety.

**Table 6:** KEGG enrichment analysis.

ID	Molecular function	*P* value	Count	Gene name
hsa04080	Neuroactive ligand-receptor interaction	2.42E-19	31	ADRA1A/ADRA1B/ADRA1D/ADRA2A/ADRA2B/ADRA2/ADRB1/ADRB2/CHRM1/CHRM2/CHRM3/CHRM4/CHRM5/CHRNA2/CHRNA7/DRD1/DRD2/EDN1/F2/GABRA1/GABRA2/GABRA3/GABRA5/GABRA6/GLRA2/GRIA2/GRIN2A/HTR2A/NR3C1/OPRD1/OPRM1
hsa04020	Calcium signaling pathway	2.39E-14	22	ADRA1A/ADRA1B/ADRA1D/ADRB1/ADRB2/CALM1/CAMK2G/CHRM1/CHRM2/CHRM3/CHRM5/CHRNA7/DRD1/GRIN2A/HTR2A/KDR/NOS1/NOS2/NOS3/PRKACA/PRKCA/VDAC1
hsa04024	cAMP signaling pathway	4.86E-10	17	ADRB1/ADRB2/AKT1/CALM1/CAMK2G/CHRM1/CHRM2/DRD1/DRD2/EDN1/GRIA2/GRIN2A/JUN/PDE4A/PDE4D/PRKACA/RELA
hsa04728	Dopaminergic synapse	2.25E-11	15	AKT1/CALM1/CAMK2G/COMT/DRD1/DRD2/GRIA2/GRIN2A/GSK3B/MAOA/MAOB/MAPK14/PRKACA/PRKCA/SLC6A3
hsa04022	cGMP-PKG signaling pathway	6.59E-10	15	ADRA1A/ADRA1B/ADRA1D/ADRA2A/ADRA2B/ADRA2C/ADRB1/ADRB2/AKT1/CALM1/NOS3/OPRD1/PDE2A/PDE5A/VDAC1
hsa05032	Morphine addiction	4.53E-10	12	DRD1/GABRA1/GABRA2/GABRA3/GABRA5/GABRA6/OPRM1/PDE2A/PDE4A/PDE4D/PRKACA/PRKCA
hsa04933	AGE-RAGE signaling pathway in diabetic complications	1.38E-09	12	AKT1/BCL2/CASP3/EDN1/IL6/JUN/MAPK14/MMP2/NOS3/PRKCA/RELA/TGFB1
hsa05031	Amphetamine addiction	3.09E-10	11	CALM1/CAMK2G/DRD1/GRIA2/GRIN2A/JUN/MAOA/MAOB/PRKACA/PRKCA/SLC6A3
hsa05030	Cocaine addiction	1.60E-10	10	DRD1/DRD2/GRIA2/GRIN2A/JUN/MAOA/MAOB/PRKACA/RELA/SLC6A3
hsa00220	Arginine biosynthesis	7.01E-11	8	ARG1/GLUL/GPT/GPT2/NOS1/NOS2/NOS3/OTC

KEGG = Kyoto encyclopedia of genes and genomes.

### 3.7. Molecular docking between components and target proteins

**Figure 14.**
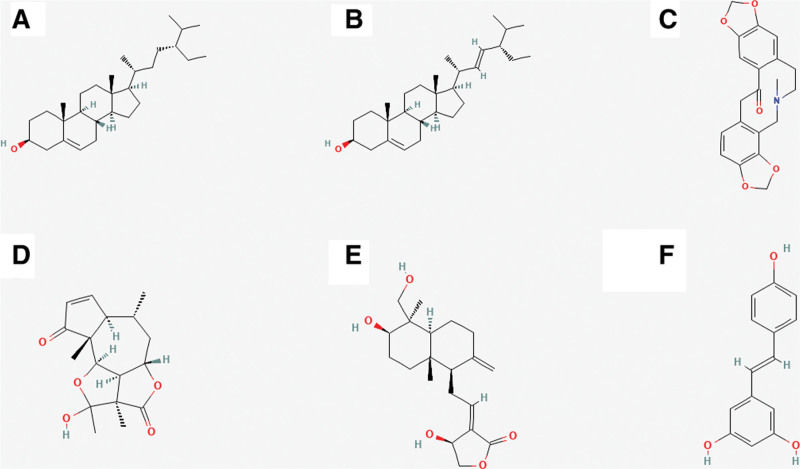
Chemical structures of core target proteins and positive drugs: (A) beta-sitosterol, (B) Stigmasterol, (C) Fumarine, (D) Tenulin, (E) Andrographolide, and (F) Resveratrol.

**Figure 15.**
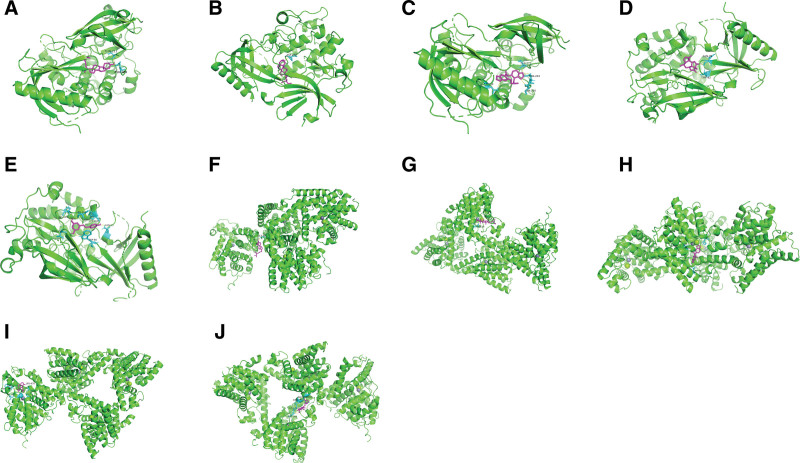
The combined figures of the 3D molecular models as followed: (A) beta-sitosterol-AKT1, (B) Stigmasterol-AKT1, (C) Fumarine-AKT1, (D) Tenulin-AKT1, (E)positive drug-AKT1, (F) beta-sitosterol-IL6, (G) Stigmasterol-IL6, (H) Fumarine-IL6, (I) Tenulin-IL6, and (J) positive drug-IL6.

**Figure 16.**
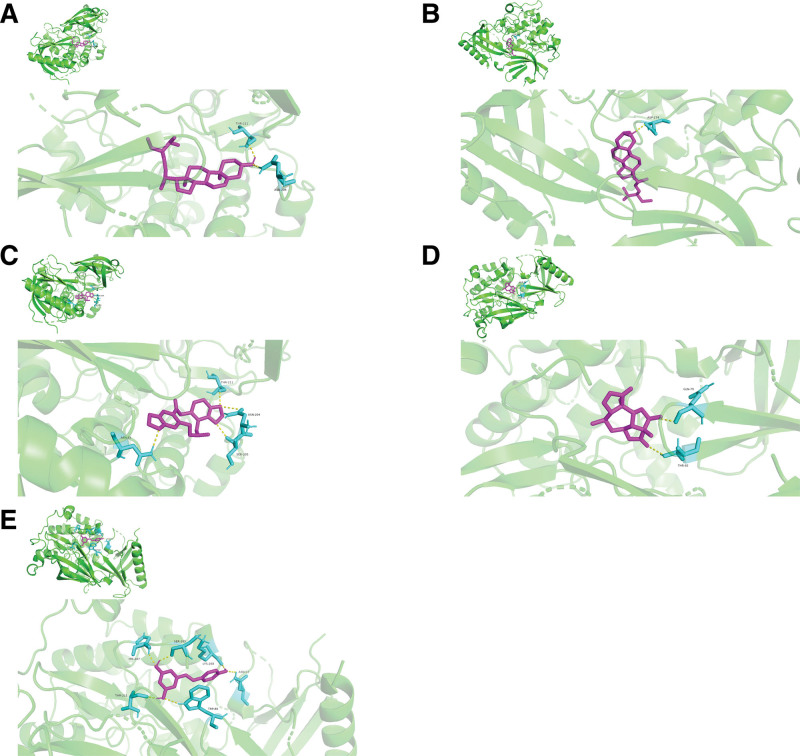
The 3D molecular models between AKT1 and drug/component as followed: (A) beta-sitosterol-AKT1, (B) Stigmasterol-AKT1, (C) Fumarine-AKT1, (D) Tenulin-AKT1, and (E) positive drug-AKT1.

**Figure 17.**
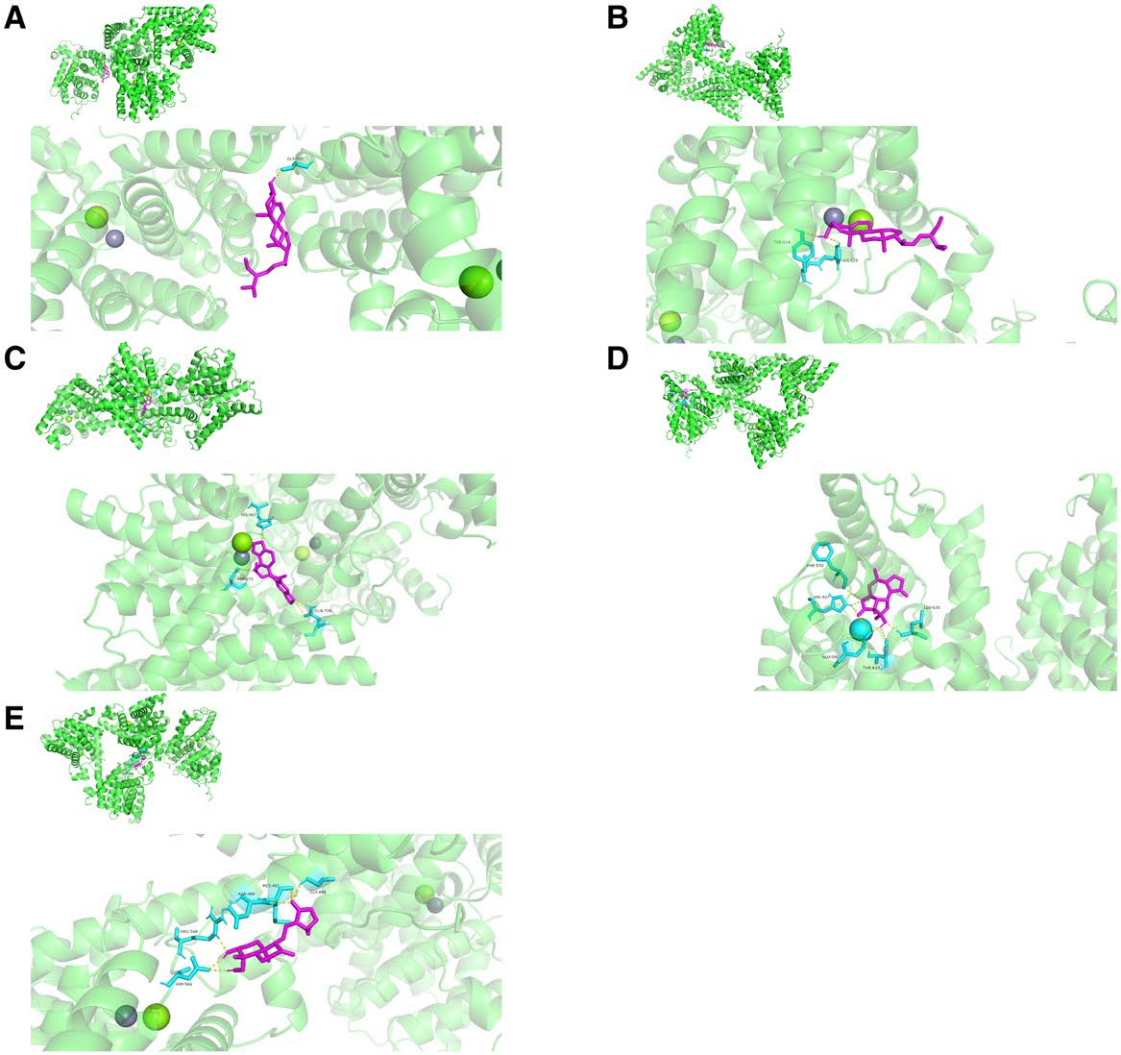
The 3D molecular models between IL-6 and drug/component as followed: (A) beta-sitosterol-IL6, (B) Stigmasterol-IL6, (C) Fumarine-IL6, (D) Tenulin-IL6, and (E) positive drug-IL6. IL = interleukin.

The 2 crucial target proteins (AKT1 and IL-6) and 4 bioactive components (MOL000449, NO1, MOL000449, and MOL000787) were selected from PPI analysis and topological analysis to conduct molecular docking. The 3D structure of AKT1 (PDB ID: 7NH5) and IL-6 (PDB ID: 5SFK) was obtained from RCSB Protein Data Bank. The ranges of binding energy for AKT1 or IL-6 which bind to each target protein were shown as follow: AKT1 (−10.88 to −6.03 kcal/mol) and IL-6 (−8.98 to −4.67 kcal/mol). After comparing with the binding energy for positive drugs (Andrographolide and Resveratrol), the results showed that each core component of KXS (beta-sitosterol, Tenulin, Stigmasterol, and Fumarine) bound to the target protein (AKT1 and IL-6) was more stable than positive drugs. The chemical structures of these bioactive components and positive drugs are shown in Figure 14. The interaction between ligands and target proteins is shown in Figure 15 (combination figure), Figure 16 (AKT1), and Figure 17 (IL-6). The lowest binding affinity for each bioactive component was shown: beta-sitosterol-AKT1 (−10.88 kcal/mol) and Fumarine-IL6 (−8.08 kcal/mol). Moreover, the lowest binding energy, the volume and size of binding pockets, and the correspondent position of amino acid residues of docking are shown in Table 7.

**Table 7:** Docking energy, the size and volume of binding pockets, and binding residues.

Protein	Ligand	Energy range (kcal/mol)	Size	Volume (A°)	Center x	Center y	Center z	Residues
AKT1	beta-sitosterol	−10.88	102 × 126 × 126	0.542	13.935	−13.229	−15.594	ASN-204 THR-211
AKT1	Stigmasterol	−9.80	106 × 126 × 122	0.525	15.118	−14.452	−15.594	ASP-274
AKT1	Fumarine	−9.73	106 × 126 × 122	0.525	15.118	−14.452	−15.594	ASN-53 ASN-204 SER-205 THR-211
AKT1	Tenulin	−7.39	96 × 116 × 126	0.575	13.935	−15.538	−15.594	GLN-79 THR-82
AKT1	positive drug	−4.79	96 × 110 × 112	0.592	13.935	−14.742	−15.594	ASN-53 TRP-80 SER-205 HIS-207 THR-211 LYS-268
IL-6	beta-sitosterol	−5.86	126 × 88 × 114	0.947	57.063	1.630	−0.069	GLY-707
IL-6	Stigmasterol	−6.61	126 × 88 × 114	0.947	57.063	1.630	−0.069	TYR-524 HIS-525
IL-6	Fumarine	−8.08	126 × 80 × 126	0.997	60.685	1.630	−0.069	HIS-567 ASP-674 GLN-726
IL-6	Tenulin	−6.43	126 × 94 × 114	0.942	57.789	0.712	−2.069	HIS-567 PHE-570 GLU-592 THR-633 LEU-635
IL-6	positive drug	−2.99	126 × 74 × 104	0.986	60.685	5.235	−2.713	ASN-484 MET-485 GLY-488 ASP-566 ARG-568

## 4. Discussion

Although KXS has been used to treat mental disorders clinically, the exact mechanisms are unclear.^[9]^ The method of combing network pharmacology study and molecular docking were used to predict the potential mechanisms of KXS treating the overlapping pathogeny of anxiety and PTSD. Sixty-four bioactive components were derived from KXS, and 3474 target genes of PTSD and 4910 target genes of anxiety were selected from several databases, respectively. The network of medicine - components - targets - disease indicated the interaction of KXS with PTSD and anxiety by multiple components and target genes.

Based on PPI network analysis, AKT1 and IL-6 might be the crucial target genes for the overlapping pathology of PTSD and anxiety. The analysis of GO enrichment indicated that overlapping genes were involved in neurotransmitter receptor activity, postsynaptic neurotransmitter receptor activity, regulation of membrane potential, vascular process in the circulatory system, localized in the synaptic membrane, and postsynaptic membrane. KEGG enrichment analysis indicated that overlapping genes were associated with neuroactive ligand-receptor interaction, calcium signaling pathway, and cAMP signaling pathway. In addition, molecular docking results indicated that beta-sitosterol and Fumarine were the crucial bioactive components, and AKT1 and IL6 were potential target genes for treating the same pathogenesis of anxiety and PTSD by KXS.

The PPI network analysis was utilized to predict a total of 103 target proteins considered the core target proteins with high degree, including AKT1, IL-6, CASP3, NOS3, and PTGS2. The AKT family of serine/threonine kinases is a crucial target protein related to multiple neuro-molecular signaling processes and has been related to neurological and psychiatric disorders. Moreover, contextual fear memory can be affected by restricted AKT1 deficiency.^[28]^ It indicated that AKT1 might be a potential target of PTSD. In addition, the hypothalamic-pituitary-adrenal (HPA) axis may be activated and the neurotransmitter metabolism may be disordered by elevated interleukin 6 (IL-6).^[29]^ A recent study showed a significant elevation of IL-6 in PTSD patients compared to healthy people.^[30]^

AKT1 is one isoform of the AKT family, studies showed that AKT1 deficiency could impact anxiety-related behavior and fear memory extinction in mice and AKT1 is modified by mood stabilizers and psychotropic medicines.^[28]^ Moreover, the crucial pathogeny of PTSD is the influence of fear memory.^[31]^ It indicated that PTSD and anxiety are associated with AKT1.

IL-6 which are often elevated in PTSD and activated the HPA axis through direct and indirect effects on corticotropin releasing hormone, adrenocorticotropin and cortisol secretion. The imbalance of the HPA axis is considered as one of the main pathogenesis of PTSD and anxiety.^[32,33]^ PPI analysis predicted that AKT1 and IL-6 might be involved in the pharmacological profile of KXS on PTSD and anxiety.

The GO enrichment analysis showed that overlapping genes were associated with numerous signal transductions in BP, MF, and CC, respectively. The results of MF and BP were associated with signaling pathways and receptors, such as neurotransmitter receptor activity, postsynaptic neurotransmitter receptor activity, G protein-coupled amine receptor activity, and regulation of membrane potential. The results of CC showed that the function or process was related to the synaptic membrane, and postsynaptic membrane in cells with target genes, including ADRA1A, ADRA2A, CHRM1, CHRM2, CHRM3, CHRM4, CHRM5, CHRNA2, CHRNA7, DRD1, DRD2, GABRA1, GABRA2, GABRA3, GABRA5, GABRA6, GLRA2, GRIA2, GRIN2A, HTR2A, HTR3A, OPRD1, OPRM1, PDE2A, SLC6A2, SLC6A3, SLC6A4, and SLC6A9.

The dysfunction of the neurotransmitter system is considered as an important pathogeny of anxiety and other mental diseases. Moreover, several neurotransmitter receptors include serotonin receptor (5-HTR), γ-aminobutyric acid (GABA) receptor, and glutamatergic receptors, etc. They are potential and pharmacological targets for anxiety and PTSD.^[34,35]^ HT4R is widely expressed in the whole central nervous system and the periphery and plays an important role in regulating emotion, anxiety, and cognition. The hippocampus specific loss of 5-HT4R will trigger anxiety-like behavior in mice. In addition, another serotonin receptor subtype is also regulated by mood, memory, anxiety, and sleep, etc.^[36,37]^ Therefore, SSRIs are often used to treat PTSD and anxiety clinically. Fear memories are learned, stored, and expunged by GABA systems. The severity of anxiety symptoms correlated with the deficit in GABAA receptors, and the study showed that patients with PTSD had obvious GABAA receptor defects in the prefrontal cortex. Therefore, ameliorating deficits in the GABA receptor and activation of GABA receptors could be a way of treating PTSD and anxiety.^[38,39]^

Besides, G protein-coupled amine receptor (GPCRs) is a considerable family of membrane receptor proteins, which widely exists on the surface of the cell membranes. GPCRs can bind to corresponding ligands to regulate the activity of intracellular enzymes and produce second messengers, thereby regulating relevant biological processes. GPCRs can not only function as monomers, but also form homodimers or heterodimers among themselves, so GPCRs are the target of numerous medications and are involved in neurological diseases.^[40,41]^ Signal proteins will trigger the anxiety-like behaviors in the GPCR pathway including Akt, p11, and MKP-1 at activating GPCRs.^[42]^ In addition, many orphan receptors are involved in numerous processes in the GPCR family, such as GPR3, GPR6, GPR16, and GPR40, etc. For instance, GPR3 plays a crucial role in various nervous system diseases and emotional reactions by regulating 5-HT and dopamine. GPR26 is found expressed in the amygdala, hippocampus, and cerebral cortex of mice and is involved in the mood regulation.^[43,44]^ Therefore, GPCRs and neurotransmitter receptors were considered as the important aspects of treating PTSD and anxiety of KXS.

The results of the KEGG enrichment analysis were also utilized to analyze potential signaling pathways for treating PTSD and anxiety by KXS. Multiple signaling pathways include neuroactive ligand-receptor interaction, calcium signaling pathway, and cAMP signaling pathway, etc. Neuroactive ligand-receptor interaction is ranked first herein and associated with neuronal processes.^[45]^ The GABA receptors and 5-HT receptors are contained in this signaling pathway and they have been discussed to play an important role in PTSD and anxiety. Especially, SSRI is a First-line medication for treating PTSD and anxiety based on the function of 5-HT receptors. Over-generalized fear is one of the main symptoms of anxiety and PTSD, but overgeneralized fear can be eliminated by blocking cholinergic muscarinic receptors, which maybe an excellent treatment for PTSD and anxiety.^[46,47]^ Glucocorticoid receptors (NR3C1) are widely expressed in the hippocampus, amygdala, and prefrontal cortex and are involved in encoding, retaining and processing information about emotional events. Especially, activation of the HPA axis will release glucocorticoids to regulate NR3C1 when the patient experiences a stressful event. But the dysfunction of NR3C1 induces anxiety and PTSD when patients are exposed to extreme events or chronic stress.^[48,49]^ kappa-opioid receptors (KORs) are a subtype of the opioid receptor family, and accumulating evidence indicates that KORs are involved in transducing the effects of stress. Many studies showed that selective KOR antagonists could resist anxiety and extinct fear.^[50,51]^

In addition, calcium signaling pathway is another important result of this KEGG enrichment analysis. Calcium is a crucial second messenger which regulates numerous biological processes. But calcium channels are variable and play an important role in synapses, the dysfunction of calcium channels and deregulation of calcium concentration can cause a series of neuropsychiatric disorders.^[52,53]^ This signaling pathway also contains the CPCR and it, which is upstream of the access road, can regulate multiple proteins/enzymes in the calcium signaling pathway. It indicated that GPCR receptors maybe a crucial target for treating PTSD and anxiety. Figure 13 shows that learning, memory, and long-term depression are indirectly affected by Ca(2+)/calmodulin-dependent protein kinase (CAMK). Medial prefrontal cortex (mPFC) is significantly involved in emotion and pain regulation, which is crucial for PTSD and anxiety, but the mPFC has plastic changes when PTSD and anxiety occur. Moreover, the expression of CAM in mPFC is significantly increased and the expression of CaMKII α was significantly reduced. And the upregulation of CaMKII can reduce anxiety-like behaviors. It indicated that dysfunction of CaM and CaMKII α might be the abnormal pathological basis.^[54,55]^ And then, calcium signaling pathway indirectly associates with other signaling pathways through CALM, NOS, and PKC, etc. The protein kinase C (PKC) plays a particularly important role in regulating the transport of GABAA receptors and its sensitivity to GABA, which are involved in the pathogenesis of anxiety and PTSD.^[56]^ Reduction of PKC phosphorylation leads to the downregulation of glucocorticoid receptor, which weakens the feedback inhibition of the HPA axis mediated by glucocorticoid receptor and promotes the stress response of the HPA axis. The HPA axis is one of the main regulatory pathways to control the physiological response to stress, so dysfunction of HPA will cause many mental diseases.^[57,58]^ Hence, calcium signaling pathway and neuroactive ligand-receptor interaction might be the crucial signal pathways involved in the pharmacological profile of KXS against PTSD and anxiety.

The interaction of proteins and ligands is usually validated by using molecular docking. The core target proteins (AKT1 and IL-6) in PPI analysis and several bioactive components with high degrees (beta-sitosterol, Tenulin, Fumarine, and Stigmasterol) were selected to conduct molecular docking. Lower binding energy means more stable binding affinity. The available screening of successful docking is −5.0 kcal/mol. To exclude false positive results, positive drugs (Andrographolide and Resveratrol) were selected to compare with the binding energy of core target proteins.^[45,59]^ The results of molecular docking showed that the binding energy of beta-sitosterol, Tenulin, Fumarine, and Stigmasterol were all lower than the positive control drugs. It indicated that beta-sitosterol, Tenulin, Fumarine, and Stigmasterol might play an important role in the treatment of PTSD and anxiety. The results which combined network pharmacological analysis and molecular docking showed that the effects of treating PTSD and anxiety of beta-sitosterol, Tenulin, Fumarine, and Stigmasterol were associated with AKT1 and IL-6.

Nevertheless, there are still many deficiencies in the research of traditional Chinese herbal medicine, such as a lot of unknown compounds, targets, and signaling pathways, so the pharmacological effects of an herbal are still incompletely clear. Moreover, there is a certain deviation in the databases of drug and disease targets due to their different methods of mining and filtering data. Increasing animal experiments are designed to confirm the corresponding results of network pharmacology study and molecular docking. Besides, modern analytical instruments and pharmacokinetic experiments will be performed to reveal the important pharmacokinetic process and corresponding signaling pathways that will clarify the effect of treating PTSD and anxiety of KXS based on the “treating different diseases with the same therapeutic principle”

## 5. Conclusion

In general, KXS was characterized by multiple bioactive components in treating PTSD and anxiety based on “treating different diseases with the same therapeutic principle.” Based on the “medicine-components-targets-diseases” network construction, bioactive components of KXS and overlapping targets of PTSD and anxiety were derived. And then, they were used to predict relevant signaling pathways (i.e., neuroactive ligand-receptor interaction, calcium signaling pathway). It indicated that KXS treated overlapping pathogeny of PTSD and anxiety through multiple components, targets, and signaling pathways. Further analysis of the pharmacological effects of KXS for treating the overlapping pathogenesis of PTSD and anxiety will be confirmed.

## Author contributions

**Conceptualization:** Wen-Wei Li, Jia Wang, Han-Biao Wu, Zhi-Kun Qiu.

**Data curation:** Wen-Wei Li, Zhi-Kun Qiu.

**Formal analysis:** Wen-Wei Li, Zhi-Kun Qiu.

**Funding acquisition:** Wen-Wei Li, Zhi-Kun Qiu.

**Investigation:** Wen-Wei Li, Zhi-Kun Qiu.

**Methodology:** Wen-Wei Li, Zhi-Kun Qiu.

**Project administration:** Wen-Wei Li, Zhi-Kun Qiu.

**Resources:** Wen-Wei Li, Zhi-Kun Qiu.

**Software:** Wen-Wei Li, Zhi-Kun Qiu.

**Supervision:** Wen-Wei Li, Zhi-Kun Qiu.

**Validation:** Wen-Wei Li, Zhi-Kun Qiu.

**Visualization:** Wen-Wei Li, Zhi-Kun Qiu.

**Writing – original draft:** Wen-Wei Li, Zhi-Kun Qiu.

**Writing – review & editing:** Wen-Wei Li, Zhi-Kun Qiu.

## References

[1] Watson P. PTSD as a public mental health priority. Curr Psychiatry Rep. 2019;21:61. 31243637 10.1007/s11920-019-1032-1

[2] Auxéméry Y. Post-traumatic psychiatric disorders: PTSD is not the only diagnosis. Presse Med. 2018;47:423–30. 29580906 10.1016/j.lpm.2017.12.006

[3] Shalev A Liberzon I Marmar C. Post-traumatic stress disorder. N Engl J Med. 2017;376:2459–69. 28636846 10.1056/NEJMra1612499

[4] Williamson J Jaffee M Jorge R. Posttraumatic stress disorder and anxiety-related conditions. Continuum. 2021;27:1738–63. 34881734 10.1212/CON.0000000000001054

[5] Abdallah CG Averill LA Akiki TJ. The neurobiology and pharmacotherapy of posttraumatic stress disorder. Annu Rev Pharmacol Toxicol. 2019;59:171–89. 30216745 10.1146/annurev-pharmtox-010818-021701 PMC6326888

[6] Kandola A Vancampfort D Herring M. Moving to beat anxiety: epidemiology and therapeutic issues with physical activity for anxiety. Curr Psychiatry Rep. 2018;20:63. 30043270 10.1007/s11920-018-0923-x PMC6061211

[7] Yeung K Hernandez M Mao J. Herbal medicine for depression and anxiety: a systematic review with assessment of potential psycho-oncologic relevance. Phytother Res. 2018;32:865–91. 29464801 10.1002/ptr.6033 PMC5938102

[8] Edinoff A Akuly H Hanna T. Selective serotonin reuptake inhibitors and adverse effects: a narrative review. Neurol Int. 2021;13:387–401. 34449705 10.3390/neurolint13030038 PMC8395812

[9] Wang J Zhou X Hu Y. Research progress on pharmacodynamic material basis and pharmacological action mechanism of Kai-Xin-San. Chin Trad Herbal Drugs. 2020;51:4780–8.

[10] Sun Y Sun T Li M. Study on modern pharmacological action and mechanism of Kaixin Powder. J Basic Chin Med. 2021;27:650–4.

[11] Teng-da Y Yu-li L Zhi-qiang T. Relation textual research between effect variation and dosage of famous classical formula Kaixinsan. Chin J Exp Trad Med Formulae. 2021;27:24–33.

[12] Yuan Z Pan Y Leng T. Progress and prospects of research ideas and methods in the network pharmacology of Traditional Chinese Medicine. J Pharm Sci. 2022;25:218–26. 10.18433/jpps32911 35760072

[13] Qiu Z Liu Z Pang J. A network pharmacology study with molecular docking to investigate the possibility of licorice against posttraumatic stress disorder. Metab Brain Dis. 2021;36:1763–77. 34417940 10.1007/s11011-021-00816-2

[14] Liu Z Guo F Wang Y. BATMAN-TCM: a bioinformatics analysis tool for molecular mechANism of Traditional Chinese Medicine. Sci Rep. 2016;6:21146. 26879404 10.1038/srep21146 PMC4754750

[15] Daina A Michielin O Zoete V. SwissADME: a free web tool to evaluate pharmacokinetics, drug-likeness and medicinal chemistry friendliness of small molecules. Sci Rep. 2017;7:42717. 28256516 10.1038/srep42717 PMC5335600

[16] Cheng H Song H Liu B. Study on the mechanism of triterpenoids from Inonotus obliquus against gastric cancer based on network pharmacology and molecular docking. Nat Product Res Dev. 2021;33:1391–400.

[17] Ru J Li P Wang J. TCMSP: a database of systems pharmacology for drug discovery from herbal medicines. J Cheminform. 2014;6:13. 24735618 10.1186/1758-2946-6-13 PMC4001360

[18] Amberger J Hamosh A. Searching online mendelian inheritance in man (OMIM): a knowledgebase of human genes and genetic phenotypes. Curr Protocols Bioinf. 2017;58:1.2.1–1.2.12. 10.1002/cpbi.27 PMC5662200 28654725

[19] Piñero J Ramírez-Anguita J Saüch-Pitarch J. The DisGeNET knowledge platform for disease genomics: 2019 update. Nucleic Acids Res. 2020;48:D845–55. 31680165 10.1093/nar/gkz1021 PMC7145631

[20] Jia A Xu L Wang Y. Venn diagrams in bioinformatics. Brief Bioinform. 2021;22:bbab108. 33839742 10.1093/bib/bbab108

[21] Doncheva NT Morris JH Gorodkin J. Cytoscape StringApp: network analysis and visualization of proteomics data. J Proteome Res. 2019;18:623–32. 30450911 10.1021/acs.jproteome.8b00702 PMC6800166

[22] Athanasios A Charalampos V Vasileios T. Protein-protein interaction (PPI) network: recent advances in drug discovery. Curr Drug Metab. 2017;18:5–10. 28889796 10.2174/138920021801170119204832

[23] Nota B. Gogadget: an R package for interpretation and visualization of GO enrichment results. Mol Inform. 2017;36. 10.1002/minf.201600132 28000438

[24] Stanzione F Giangreco I Cole JC. Use of molecular docking computational tools in drug discovery. Prog Med Chem. 2021;60:273–343. 34147204 10.1016/bs.pmch.2021.01.004

[25] Forli S Huey R Pique ME. Computational protein-ligand docking and virtual drug screening with the AutoDock suite. Nat Protoc. 2016;11:905–19. 27077332 10.1038/nprot.2016.051 PMC4868550

[26] Xiao YG Wu HB Chen JS. Exploring the potential antidepressant mechanisms of Pinellia by using the network pharmacology and molecular docking. Metab Brain Dis. 2022;37:1071–94. 35230627 10.1007/s11011-022-00930-9

[27] Seeliger D de Groot BL. Ligand docking and binding site analysis with PyMOL and Autodock/Vina. J Comput Aided Mol Des. 2010;24:417–22. 20401516 10.1007/s10822-010-9352-6 PMC2881210

[28] Wong H Levenga J LaPlante L. Isoform-specific roles for AKT in affective behavior, spatial memory, and extinction related to psychiatric disorders. eLife. 2020;9:e56630. 33325370 10.7554/eLife.56630 PMC7787664

[29] Ting E Yang A Tsai S. Role of interleukin-6 in depressive disorder. Int J Mol Sci . 2020;21. 10.3390/ijms21062194 PMC7139933 32235786

[30] Rhein C Hepp T Kraus O. Interleukin-6 secretion upon acute psychosocial stress as a potential predictor of psychotherapy outcome in posttraumatic stress disorder. J Neural Transmission. 2021;128:1301–10. 10.1007/s00702-021-02346-8 33988765

[31] Kirkpatrick H Heller G. Post-traumatic stress disorder: theory and treatment update. Int J Psychiatry Med. 2014;47:337–46. 25084856 10.2190/PM.47.4.h

[32] Frankiensztajn L Elliott E Koren O. The microbiota and the hypothalamus-pituitary-adrenocortical (HPA) axis, implications for anxiety and stress disorders. Curr Opin Neurobiol. 2020;62:76–82. 31972462 10.1016/j.conb.2019.12.003

[33] Dunlop B Wong A. The hypothalamic-pituitary-adrenal axis in PTSD: Pathophysiology and treatment interventions. Progr Neuro Psychopharmacol Biol Psychiatr. 2019;89:361–79. 10.1016/j.pnpbp.2018.10.010 30342071

[34] Olivier J Olivier B. Translational studies in the complex role of neurotransmitter systems in anxiety and anxiety disorders. Adv Exp Med Biol. 2020;1191:121–40. 32002926 10.1007/978-981-32-9705-0_8

[35] Rasmusson A Pineles S. Neurotransmitter, peptide, and steroid hormone abnormalities in PTSD: biological endophenotypes relevant to treatment. Curr Psychiatry Rep. 2018;20:52. 30019147 10.1007/s11920-018-0908-9

[36] Karayol R Medrihan L Warner-Schmidt J. Serotonin receptor 4 in the hippocampus modulates mood and anxiety. Mol Psychiatry. 2021;26:2334–49. 33441982 10.1038/s41380-020-00994-y PMC8275670

[37] Pytliak M Vargová V Mechírová V. Serotonin receptors – from molecular biology to clinical applications. Physiol Res. 2011;60:15–25. 20945968 10.33549/physiolres.931903

[38] Möhler H. The GABA system in anxiety and depression and its therapeutic potential. Neuropharmacology. 2012;62:42–53. 21889518 10.1016/j.neuropharm.2011.08.040

[39] Jembrek MJ Vlainic J. GABA receptors: pharmacological potential and pitfalls. Curr Pharm Des. 2015;21:4943–59. 26365137 10.2174/1381612821666150914121624

[40] Patwardhan A Cheng N Trejo J. Post-translational modifications of G protein-coupled receptors control cellular signaling dynamics in space and time. Pharmacol Rev. 2021;73:120–51. 33268549 10.1124/pharmrev.120.000082 PMC7736832

[41] Duc N Kim H Chung K. Structural mechanism of G protein activation by G protein-coupled receptor. Eur J Pharmacol. 2015;763:214–22. 25981300 10.1016/j.ejphar.2015.05.016

[42] Hauger R Olivares-Reyes J Dautzenberg F. Molecular and cell signaling targets for PTSD pathophysiology and pharmacotherapy. Neuropharmacology. 2012;62:705–14. 22122881 10.1016/j.neuropharm.2011.11.007 PMC3545643

[43] Alavi M Shamsizadeh A Azhdari-Zarmehri H. Orphan G protein-coupled receptors: the role in CNS disorders. Biomed Pharmacother. 2018;98:222–32. 29268243 10.1016/j.biopha.2017.12.056

[44] Civelli O. Orphan GPCRs and neuromodulation. Neuron. 2012;76:12–21. 23040803 10.1016/j.neuron.2012.09.009 PMC3474844

[45] Wu H Xiao Y Chen J. The potential mechanism of Bupleurum against anxiety was predicted by network pharmacology study and molecular docking. Metab Brain Dis. 2022;37:1609–39. 35366129 10.1007/s11011-022-00970-1

[46] Rafiq S Batool Z Liaquat L. Blockade of muscarinic receptors impairs reconsolidation of older fear memory by decreasing cholinergic neurotransmission: a study in rat model of PTSD. Life Sci. 2020;256:118014. 32593712 10.1016/j.lfs.2020.118014

[47] Fuenzalida M Pérez M Arias H. Role of nicotinic and muscarinic receptors on synaptic plasticity and neurological diseases. Curr Pharm Des. 2016;22:2004–14. 26818867 10.2174/1381612822666160127112021

[48] Finsterwald C Alberini C. Stress and glucocorticoid receptor-dependent mechanisms in long-term memory: from adaptive responses to psychopathologies. Neurobiol Learn Mem. 2014;112:17–29. 24113652 10.1016/j.nlm.2013.09.017 PMC3979509

[49] Miao X Chen Q Wei K. Posttraumatic stress disorder: from diagnosis to prevention. Mil Med Res. 2018;5:32. 30261912 10.1186/s40779-018-0179-0 PMC6161419

[50] Van’t Veer A Carlezon W. Role of kappa-opioid receptors in stress and anxiety-related behavior. Psychopharmacology (Berl). 2013;229:435–52. 23836029 10.1007/s00213-013-3195-5 PMC3770816

[51] Jacobson M Wulf H Browne C. The kappa opioid receptor antagonist aticaprant reverses behavioral effects from unpredictable chronic mild stress in male mice. Psychopharmacology (Berl). 2020;237:3715–28. 32894343 10.1007/s00213-020-05649-y PMC7686052

[52] Brini M Calì T Ottolini D. Neuronal calcium signaling: function and dysfunction. Cell Mol Life Sci. 2014;71:2787–814. 24442513 10.1007/s00018-013-1550-7 PMC11113927

[53] Nanou E Catterall W. Calcium channels, synaptic plasticity, and neuropsychiatric disease. Neuron. 2018;98:466–81. 29723500 10.1016/j.neuron.2018.03.017

[54] Wen Y Li B Han F. Dysfunction of calcium/calmodulin/CaM kinase IIα cascades in the medial prefrontal cortex in post-traumatic stress disorder. Mol Med Rep. 2012;6:1140–4. 22895536 10.3892/mmr.2012.1022

[55] Cao J Liu X Liu J. Inhibition of glutamatergic neurons in layer II/III of the medial prefrontal cortex alleviates paclitaxel-induced neuropathic pain and anxiety. Eur J Pharmacol. 2022;936:175351. 36309050 10.1016/j.ejphar.2022.175351

[56] Nakamura Y Darnieder L Deeb T. Regulation of GABAARs by phosphorylation. Adv Pharmacol. 2015;72:97–146. 25600368 10.1016/bs.apha.2014.11.008 PMC5337123

[57] Di T Zhang S Hong J. Hyperactivity of hypothalamic-pituitary-adrenal axis due to dysfunction of the hypothalamic glucocorticoid receptor in sigma-1 receptor knockout mice. Front Mol Neurosci. 2017;10:287. 28932185 10.3389/fnmol.2017.00287 PMC5592243

[58] Sbarski B Akirav I. Cannabinoids as therapeutics for PTSD. Pharmacol Therap. 2020;211:107551. 32311373 10.1016/j.pharmthera.2020.107551

[59] Yin B Bi YM Fan GJ. Molecular mechanism of the effect of Huanglian Jiedu decoction on type 2 diabetes mellitus based on network pharmacology and molecular docking. J Diabetes Res. 2020;2020:5273914. 33134394 10.1155/2020/5273914 PMC7593729

